# Mutanlallemand (*mtl*) and Belly Spot and Deafness (*bsd*) Are Two New Mutations of *Lmx1a* Causing Severe Cochlear and Vestibular Defects

**DOI:** 10.1371/journal.pone.0051065

**Published:** 2012-11-30

**Authors:** Georg Steffes, Beatriz Lorente-Cánovas, Selina Pearson, Rachael H. Brooker, Sarah Spiden, Amy E. Kiernan, Jean-Louis Guénet, Karen P. Steel

**Affiliations:** 1 Wellcome Trust Sanger Institute, Hinxton, United Kingdom; 2 Wolfson Centre for Age-Related Diseases, King’s College London, United Kingdom; 3 MRC Institute of Hearing Research, Nottingham, United Kingdom; 4 University of Rochester Medical Center, Rochester, New York, New York, United States of America; 5 Institut Pasteur, Paris, France; IGBMC/ICS, France

## Abstract

*Mutanlallemand* (*mtl*) and *Belly Spot and Deafness* (*bsd*) are two new spontaneous alleles of the *Lmx1a* gene in mice. Homozygous mutants show head tossing and circling behaviour, indicative of vestibular defects, and they have short tails and white belly patches of variable size. The analysis of auditory brainstem responses (ABR) showed that *mtl* and *bsd* homozygotes are deaf, whereas heterozygous and wildtype littermates have normal hearing. Paint-filled inner ears at E16.5 revealed that *mtl* and *bsd* homozygotes lack endolymphatic ducts and semicircular canals and have short cochlear ducts. These new alleles show similarities with *dreher* (*Lmx1a*) mutants. Complementation tests between *mtl* and *dreher* and between *mtl* and *bsd* suggest that *mtl* and *bsd* are new mutant alleles of the *Lmx1a* gene. To determine the *Lmx1a* mutation in *mtl* and *bsd* mutant mice we performed PCR followed by sequencing of genomic DNA and cDNA. The *mtl* mutation is a single point mutation in the 3′ splice site of exon 4 leading to an exon extension and the activation of a cryptic splice site 44 base pairs downstream, whereas the *bsd* mutation is a genomic deletion that includes exon 3. Both mutations lead to a truncated LMX1A protein affecting the homeodomain (*mtl*) or LIM2-domain (*bsd*), which is critical for LMX1A protein function. Moreover, the levels of *Lmx1a* transcript in *mtl* and *bsd* mutants are significantly down-regulated. *Hmx2/3* and *Pax2* expression are also down-regulated in *mtl* and *bsd* mutants, suggesting a role of *Lmx1a* upstream of these transcription factors in early inner ear morphogenesis. We have found that these mutants develop sensory patches although they are misshapen. The characterization of these two new *Lmx1a* alleles highlights the critical role of this gene in the development of the cochlea and vestibular system.

## Introduction

The mammalian inner ear is divided into auditory and vestibular parts. The auditory part is the cochlea, responsible for detection of sound, whereas the vestibular part comprises three semicircular canals that allow detection of head movement, and a utricle and a saccule, essential for detection of gravity and balance. The inner ear derives from the ectodermal placodes, located on either side of the hindbrain at the level of rhombomeres 5 and 6. These otic placodes invaginate and eventually close to form the otocyst. Throughout development the otocyst undergoes a series of morphogenetic events leading to the development of the different components of the inner ear.

Here we report two new spontaneous mutants, *mutanlallemand* (*mtl*), first identified in a colony at the Institut Pasteur, and *belly spot and deafness* (*bsd*), which arose in a colony at the Sanger Institute. *mtl* and *bsd* homozygotes display a similar phenotype, showing circling, head-bobbing and hyperactivity, which indicate inner ear abnormalities. Homozygotes also have short tails and white belly patches of variable size. The mutants lack a Preyer reflex (ear flick in response to noise) indicating a severe hearing impairment. Homozygous *mtl* females have no uterus (Yvan Lallemand, personal communication).

These defects are strongly reminiscent of the now extinct *shaker-short* mutants [1], [2] and of *dreher* (*dr*) mutants [3]. The disruption of the *Lmx1a* gene locus by *dreher* mutations was first reported for *Lmx1a^dr-J^*, *Lmx1a^dr-sst^*, *Lmx1a^dr-3J^* and *Lmx1a^dr-4J^* [4] and later for more alleles, including *Lmx1a^dr-2J^* [5] Previous complementation analysis demonstrated allelism between *Lmx1a^dr-2J^* and the original *dreher* (*Lmx1a^dr^* [6]). To date 13 *Lmx1a* spontaneous alleles have been reported (MGI: 1888519). A rat mutation of *Lmx1a* with short tail and head bobbing has also been reported [7]. The complementation tests we performed suggest that we have identified two new *Lmx1a* alleles.

LMX1A is a LIM homeodomain transcription factor containing two LIM domains and a homeodomain. The molecular analysis of most of the known *Lmx1a* alleles revealed point mutations affecting each of the LIM domains and deletions with/without resulting frameshift mutations affecting LIM domains and/or homeodomain, indicating that those domains are essential for proper function of LMX1A protein [5].

*Lmx1a* mRNA is expressed from embryonic day 8.5 (E8.5) [8] during development of the CNS, and has been shown to be essential for controlling the formation of the CNS roof plate [4]. *Lmx1a* is also expressed in the otic vesicle, cerebellum and neural crest cells [4], [8]. Extensive studies have looked at the role of this gene in the development of the brain, and in particular the cytoarchitecture of the cerebellum [4], [5], [9], [10], [11], [12], but only a few studies have focused on the role of *Lmx1a* in morphogenesis of the developing inner ear [13], [14] and for those studies *Lmx1a^dr-J^* was the *Lmx1a* allele analysed.

The initial descriptions of *shaker-short* (already extinct) by Bonnevie [2] reported that these mice were deaf, had short tails and showed severe disturbances of movement. In embryos the morphological abnormalities close to the dorsal midline of the myelencephalon seemed to precede the otic vesicle defects during development. The absence of endolymphatic duct and semicircular canals in those mutant mice was documented as well. Later studies by Deol [15] suggested that also in *Lmx1a^dr^* the same neural defect precedes the otic defects by at least one day of development.

Overall, the defects in *shaker-short* resulted in a disorganized cyst-like vestibular part of the inner ear replacing the semicircular canals, utricle and saccule, lack of endolymphatic sac/duct and an “abortive” organ of Corti. Similarly, classic studies from Fischer [16], [17], [18] reported inner ear morphological defects of *dreher* mutants (*Lmx1a^dr^*), showing a widened and joint utriculo-saccular space, however he observed great variability for semicircular canal morphology phenotypes and also for the cochlear morphology. Regarding this latter phenotype Fischer described severe phenotypes at postnatal ages with cochleae missing scala vestibuli as well as more moderate forms where scala tympani and scala vestibuli are well differentiated, but where the perilymphatic space is expanded at the expense of the endolymphatic space [17].

Due to similarities in the phenotype and behaviour of *mtl* and *bsd* mutants with *shaker-short* and *dreher* mutants we analyzed the hearing function and inner ear morphology of these two new mutants and we demonstrate that *mtl* and *bsd* are two new *Lmx1a* alleles.

## Materials and Methods

### Ethics Statement

The care and use of animals was carried out in full compliance with UK Home Office regulations and under the authorization of a Home Office Project License, approved by the local Sanger Institute Ethical Review Committee. ABR was carried out on mice fully anaesthetized with ketamine/xylazine and mice were culled using appropriate and approved humane methods.

### Mice

*Mutanlallemand* (*mtl*) is a random spontaneous mutation initially discovered by Yvan Lallemand at the Institut Pasteur, Paris, in the course of a gene targeting experiment, but the *mtl* phenotype was not linked to the transgenic insertion [19]. The genetic background of the strain is mixed. For the complementation test *Lmx1a^dr-J^* mice were used. *Belly spot and deafness* (*bsd*) arose as a recessive spontaneous mutation on a 129S5 genetic background at the Wellcome Trust Sanger Institute. To assess allelism with *mtl* we performed a complementation test crossing +/*mtl* x +/*bsd* and the offspring were analysed for the presence of any behavioral defects (circling, head-bobbing) and white belly patches. In addition we measured the length of the tails and hearing function was tested by ABR (see below). Like other *Lmx1a* alleles [20], *mtl* homozygous females have no uterus (Yvan Lallemand, personal communication). While *mtl* was maintained by crossing +/*mtl* x +/*mtl*, *bsd* were maintained crossing +/*bsd* x +/+ matings, and +/*bsd* x +/*bsd* matings were used to produce the homozygotes for analysis. Embryos were taken from timed-matings, and the day of the vaginal plug was considered day 0.5. Age-matched wild-type littermates were used as controls. They were also staged on the basis of morphology upon collection [21], [22].

### Auditory Physiology

Hearing was tested using the Auditory Brainstem Response (ABR). Mice were anaesthetized with an intraperitoneal injection of ketamine (Ketaset; 100 mg/kg) and xylazine (Rompun; 10 mg/kg). Mice were placed on a heating pad maintained at 38°C inside a sound attenuating cabinet. Subcutaneous electrodes were inserted into the skin overlying the bullae on the left (reference) and the right (ground) side of the head and at the vertex (active) to record responses to free-field stimuli. The mouse was placed so its interaural axis was 20 cm from the front edge of the speaker. The sound delivery system was calibrated with an ACO Pacific 7017 microphone. The stimuli used to evoke ABRs were generated using custom written software and Tucker Davis Technologies (TDT) hardware. Clicks of 10 µs duration were presented from 0–95 dB sound pressure level (SPL) in 5 dB steps. 5 ms pure tone pips of 6, 12, 18, 24 and 30 kHz, with a 1 ms rise/fall time, were presented in 5 dB SPL steps. The stimuli were presented 256 times at 42.6/s and averaged using custom software. ABR thresholds were determined offline by identifying the lowest sound intensity at which an ABR response could be visually identified. To facilitate recovery from anaesthetic following ABR recordings, mice were given an intraperitoneal injection of atipamezole hydrochloride (antisedan; 1 mg/kg). Detailed methods have been reported elsewhere [23].

### Morphology of the Ear

Middle ear dissections were carried out on fresh tissue (n = 1+/+, and n = 2 *bsd/bsd*) at 8 weeks old and ossicles were dissected, fixed and examined under a dissecting microscope. Observations were recorded using a standard tick sheet as described previously [24]. For the analysis of the gross morphology inner ear clearing was performed using glycerol as previously described [25] (n = 1+/+ and n = 2 *bsd/bsd*).

Paint-filling of the inner ear was performed as described [26], [27] (n = 10+/+, n = 5 *bsd/bsd*, n = 5 *mtl/mtl*). The ears were subsequently dissected out under a dissecting microscope for analysis and photographs were taken.

### Immunofluorescence

For dissections exposing sensory epithelia in whole mount inner ear/otocyst and for cochlear surface preparations of the organ of Corti from new born mice (P0) heads were bisected and inner ears plus surrounding bone were removed from the skull and then fixed in 4% paraformaldehyde for 2 hours at room temperature. Subsequently specimens were fine dissected in PBS, then washed and permeabilized in 1% PBS/Triton-X-100 (PBT) and blocked with 10% sheep serum. Then, they were incubated with the primary antibody, rabbit polyclonal against Myo7a (Proteus, cat. no. 25-6790, dilution 1∶1000 ) overnight at 4°C. After washes with PBT, samples were incubated with anti-rabbit Alexa Fluor 488 secondary antibody (Invitrogen, anti-rabbit, diluted 1∶300) and rhodamine/phalloidin (Invitrogen, diluted 1∶200). Samples were mounted in Vectashield mounting medium with DAPI (Vector Lab, H-1200). When necessary and for better 3D preservation, specimens were mounted in microscope cavity slides (Marienfeld Lauda- Königshofen, Germany). Images were acquired on a LSM 510 Meta confocal microscope (Zeiss, Welwyn Garden City). Post-acquisition image analyses were performed using Volocity3D Image Analysis software (PerkinElmer) and Adobe Photoshop CS2.

### Genotyping of *mtl* and *bsd* Mice

Genomic DNA was extracted from ear/tail biopsies or yolk sac from embryos, following standard protocols. To genotype *mtl* and *bsd* mice, *Lmx1a* exon 4 was amplified by PCR followed by sequencing. Primers were: forward 5′-AGAGCCTTTGCAAGTCAGC-3′ and reverse 5′-TGTTTGTGAGCCAGGAGTTG-3′. The amplified region encompassed exon 4 plus 220 base pairs downstream of exon 4 within intron 4/5 (amplicon size of 350 base pairs). To genotype *mtl* mice the sequence analysis identified a single G to A base pair change in the 3′ splice site of *Lmx1a* exon 4 of transcript Lmx1a-001 (ENSMUST00000028003). To genotype *bsd* mice we identified a single nucleotide polymorphism (SNP) at 34 base pairs downstream of exon 4, within intron 4/5. This single A to G base pair change, which segregates with the phenotype, was used to genotype these mice. This variation is deposited in SNPdb as rs49997218 and is not exclusive to *bsd* mice.

### Real-time PCR

Whole embryos and yolk sacs were dissected at embryonic day 10.5 (E10.5, n = 10+/+, n = 5 *bsd/bsd*, n = 5 *mtl/mtl*). DNA from yolk sacs was extracted and PCR was performed and sequenced for genotyping. Total RNA from whole embryos was isolated using RNeasy Mini Kit (Qiagen, Valencia, CA, USA) following manufacturer’s instructions. The normalized levels of RNA were used for reverse transcription using DNase I (Sigma, UK) to remove any trace of DNA contamination, followed by purification and reverse-transcription to cDNA using Superscript II-reverse transcriptase (Invitrogen, Carlsbad, CA, USA). Quantitative Real Time PCR (qRT-PCR) was carried out on an ABI Prism 7000 Sequence Detection System (Applied Biosystems) using TaqMan reagents and probes for: *Lmx1a* (Mm00473947_m1), *Hprt* (Mm01318747_g1), *Hmx3* (Mm00433957_g1), *Hmx2* (Mm01318747), *Netrin1* (Mm00500896) and *Pax2* (Mm01217939). Reagents were incubated at 50°C for 2 min followed by 90°C for 10 min. The PCR products were analysed over 40 cycles of 95°C for 15 sec followed by 60°C for 30 sec. We used *Hprt* (a house keeping gene) as an indication of the quantity of tissue compared. Levels of transcript were normalized to *Hprt* transcript. Each sample was run in triplicate and compared to littermates. The estimated gene expression was calculated using 2^−ΔΔCt^ analysis [28]. Statistical significance was estimated using the Student’s *t* test to compare mean values.

### *In situ* Hybridization

The *Lmx1a in situ* hybridization probe was generated by RT-PCR on cDNA from wildtype E10.5 embryos. Primers were designed using Primer3 software (http://frodo.wi.mit.edu/primer3/). Specific PCR primers for mouse *Lmx1a* were: *Lmx1a*-Forward-5′-AGGATACCTGGGACCACAGAGCAGG-3′ and *Lmx1a*-Reverse-5′-TACACCCGCCTCATTCTCAGCCC-3′. Forward and reverse primers were coupled to T7 and T3 promoter sequences, respectively. Digoxygenin-labelled RNA probes were generated by *in vitro* transcription using T3 and T7 polymerases. Embryos from timed matings were collected at E10.5 and E12.5. For whole mount *in situ* hybridization, embryos were fixed overnight at 4°C in 4% paraformaldehyde in PBS. Samples were embedded in paraffin and cut into 8 µm sections and hybridized using the Ventana Discovery system (Ventana Medical Systems, Inc. Illkirch, France) according to manufacturer’s instructions.

### Immunohistochemistry

Embryos were dissected in PBS, fixed for 48 hours at 4°C in 10% neutral-buffered formalin, embedded in paraffin and cut into 8 µm sections and the Ventana Discovery system (Ventana Medical Systems, Inc. Illkirch, France) was used to label according to the manufacturer’s instructions. The primary antibodies used were anti-Sox2 (1∶50, AbCam, cat. no. ab15830), anti-Jag1 (1∶50, Santa Cruz, cat. no.sc-6011), anti-p27^Kip1^ (1∶50, Cell Signalling, cat. no. 2552), anti-Myo7a (1∶400, Proteus, cat. no. 25-6790), anti-Prox1 (1∶400, Chemicon International, cat. no. ab5475), anti-FOXI1 (1∶100, AbCam, cat. no. 20454), anti-FGF9 (1∶75, AbCam, cat. no. ab9743), anti-Pax2 (1∶100, Abcam, cat. no. ab37129), anti-Netrin1 (1∶50, Alexis Biochemicals, cat. ALX-210-943) and anti-Hmx3 (1∶50, LSBios, cat. no. LS-C100622). The secondary antibodies used were anti-goat (Jackson ImmunoResearch, cat.no. 705-065-147) and anti-rabbit (Jackson ImmunoResearch, cat.no. 711-065-152), diluted 1∶100.

## Results

### Origin of *mtl* and *bsd* Mutations and Phenotype

**Figure 1:**
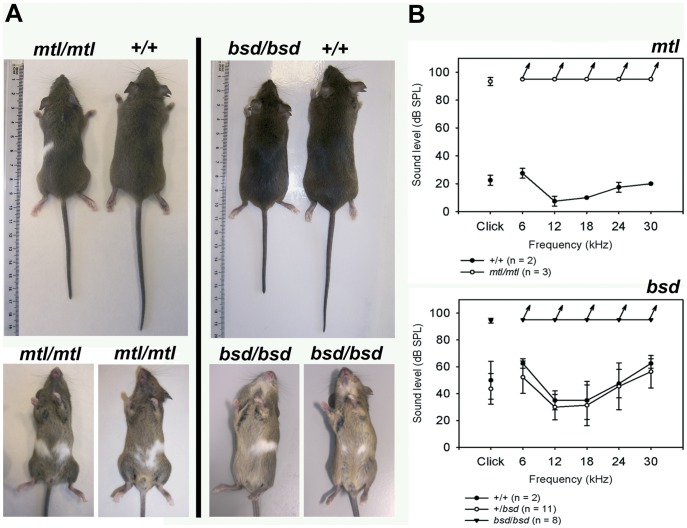
Phenotype and hearing sensitivity of *mtl* and *bsd* adult mice. A , Dorsal views show the different body size and tail lengths of wildtype and homozygotes. Ventral views show the presence of white belly patches of variable size in *mtl* and *bsd* homozygotes. **B**, Mean ABR thresholds (± standard deviation) plots for *mtl*/*mtl* compared to +/+ tested at 6–7 weeks of age (upper panel) and plots showing the mean ABR thresholds of mice with genotypes +/+, +/*bsd* and *bsd*/*bsd* around 6 weeks of age (lower panel). Clicks and tones were presented up to 95 dB SPL; all mutants showed no detectable ABR at even the highest sound level presented (these values are indicated by the arrows). The absence of discernible ABR at 95 dB SPL suggests a profound hearing loss.

*Mutanlallemand* (*mtl*) is a spontaneous recessive mutation identified in a colony at the Institut Pasteur. The mutation arose on a 129S5 genetic background, but is now maintained on a mixed background. Mutants display hyperactivity, head-tossing, circling and poor righting reflex and reaching response. These behavioural defects are indicative of an inner ear abnormality. Homozygotes also show pigmentation defects, displaying a white belly patch of variable size, and they have short or blunt tails compared to their control littermates (Fig. 1A).

*Belly Spot and Deafness* (*bsd*) is another spontaneous mutation identified at the Sanger Institute. This mutation arose in a 129S5 genetic background and homozygotes display a phenotype similar to *mtl* mutants (Fig. 1A).

Both *mtl* and *bsd* homozygotes lack a Preyer reflex (ear flick in response to noise) suggesting a severe hearing impairment. To further test the hearing sensitivity of these mice we performed auditory brainstem response (ABR) threshold measurements. Our results indicate that both *mtl* and *bsd* homozygotes showed no ABR response at 95 dB SPL (highest sound level presented) in all frequencies tested, compared to their control littermates (Fig. 1B) suggesting a severe hearing impairment.

### Complementation Tests Suggest Two New *Lmx1a* Alleles

Taken together, the phenotypic defects shown by *mtl* and *bsd* homozygous mice are strongly reminiscent of the allelic series of mutants initially founded by *dreher* [3], affecting the *Lmx1a* genomic locus on mouse chromosome 1 [4]. To determine if these mutants are novel *Lmx1a* alleles we performed two complementation tests. We started by crossing +/*Lmx1a^dr-J^* with +/*mtl*. The progeny were screened for the presence of any behavioural abnormality, pigmentation defect, short tails and lack of Preyer reflex, as in *Lmx1a^dr-J^* and *mtl* homozygotes. In the analysis of the offspring we identified mice showing the affected phenotype (Table 1) suggesting non-complementation of these two mutants.

**Table 1:** Complementation tests *dr^J^/mtl* and *bsd/mtl.*

Complementation test	Affected mice	Total number
***mtl/dr^J^***	**16**	**52**
***bsd/mtl***	**7**	**24**

**Figure 2:**
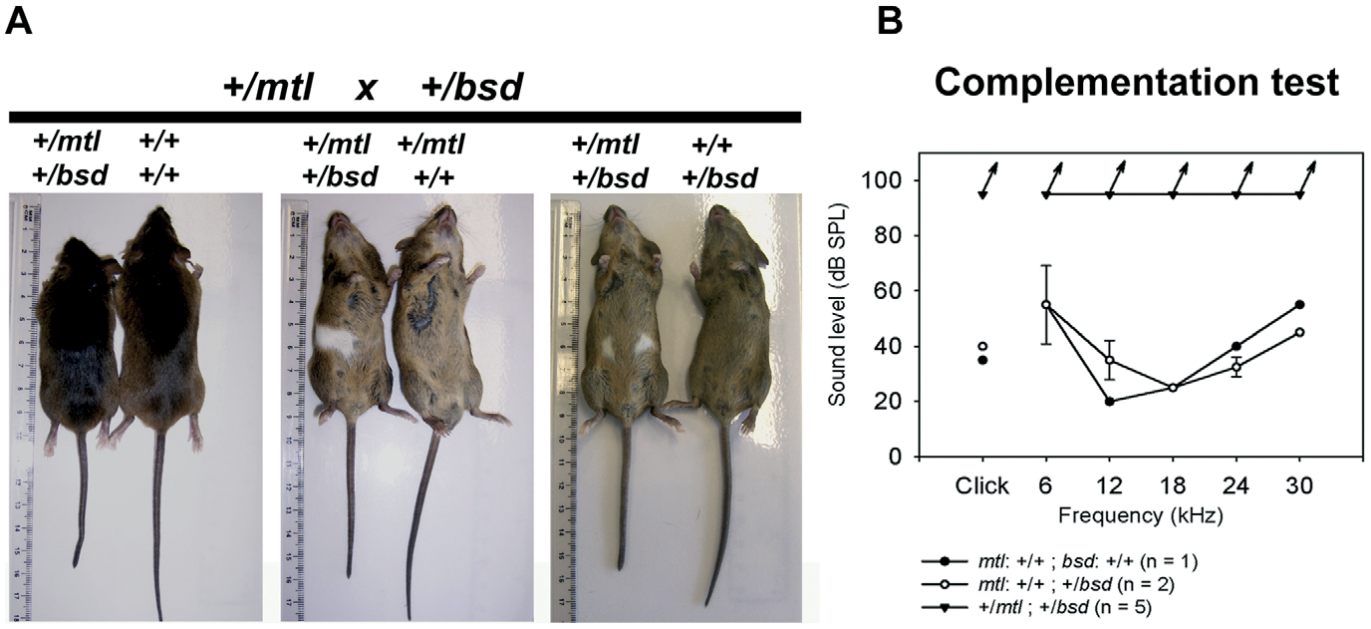
Complementation test between *mtl* and *bsd* mice. A , Phenotype of *mtl* and *bsd* adult mice from the complementation tests. Dorsal and ventral views are shown and genotypes indicated. We found mice which were compound mutants *mtl*/*bsd* showing the affected phenotype, shorter tails and white belly patches, whereas other mice were +/+, +/*mtl* or +/*bsd* and showed wildtype phenotype. **B**, Mean ABR thresholds (± standard deviation) are plotted for the complementation test at 16 weeks old. Clicks and tones were presented up to 95 dB SPL; all mice showed no detectable ABR at even the highest sound level presented (these values are indicated by the arrows), which suggests a profound hearing loss.

Similarly, to check whether *bsd* was a new *Lmx1a* allele we set up a complementation test crossing +/*bsd* and +/*mtl*. In the offspring we found both affected (compound heterozygotes, *mtl*/*bsd*) and unaffected mice (+/+, +/*bsd* or +/*mtl*) (Table 1), suggesting non-complementation of these two alleles (Fig. 2A). We also checked the hearing sensitivity of the mice derived from this complementation test by ABR and we found mice which were hearing impaired, showing the affected phenotype and genotyped as compound heterozygotes (*mtl*/*bsd*) (Fig. 2B).

### *Lmx1a* Gene is Mutated in *mtl* and *bsd* Mutant Mice

To identify the mutation in the *Lmx1a* gene of *mtl* and *bsd* mice we analysed the genomic and transcript sequence of *Lmx1a* and we compared with control mice (list of primers in Tables S1 and S2). *Lmx1a* (LIM homeobox transcription factor 1 alpha gene, MGI: 1888519, ENSMUSG00000026686; according to NCBIM37, Ensembl release 65) is located on mouse chromosome 1 and there are two different transcripts described for this gene, Lmx1a-001 (ENSMUST00000028003; 3346 base pairs in 8 exons) and Lmx1a-002 (ENSMUST00000111377; 3198 base pairs in 9 exons). Both transcripts are translated into an identical protein of 382 amino acids. The difference between the two transcripts is the arrangement of exon 1 and 5′UTR, the rest of the exons are similar in length and sequence, although they are named differently. Thus, exon 2 in transcript Lmx1a-001 is exon 3 in transcript Lmx1a-002, and so on.

**Figure 3:**
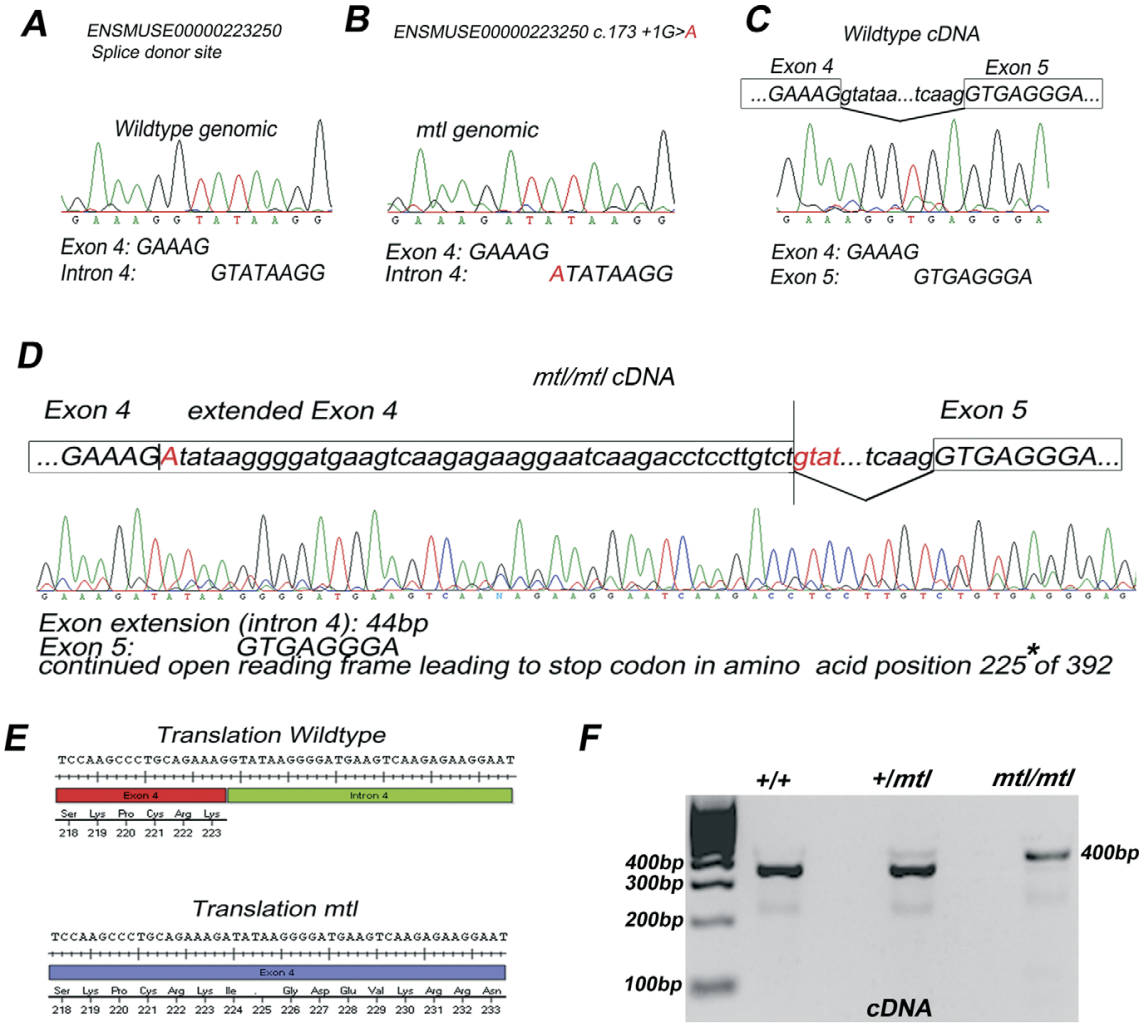
A point mutation in *mtl* mutants leads to a splice defect. A , Genomic sequence of the splice donor site at 3′ end of exon 4 of transcript Lmx1a-001. **B**, Base pair change c.173+1 G>A identified in *mtl* mutants. **C**, **D**, Partial traces and sequence of cDNA obtained from E10.5 whole embryos. **C**, Wildtype transcript cDNA pre and post splicing, exonic sequence in upper case and intronic sequence in lower case. The sequence trace shows cDNA of spliced transcript at the 3′ end of exon 4 and 5′ end of exon 5. **D**, cDNA from *mtl* mutant showing the irregular splicing between exons 4 and 5, causing an extension of 44 base pairs into intron 4 (sequence in lower case). This extension ends as it finds a cryptic splice acceptor site, GTAT (in red colour), within intron 4. **E**, Effect of the altered transcript is a continued open reading frame in *mtl* homozygotes of the exon 4 leading to a termination of protein translation at the TAA stop codon in the altered amino acid position 225* (arrow). **F**, PCR amplification of cDNA using primers located within exon 3 and exon 6. Bands of the proper size are amplified for wildtype and heterozygotes whereas in *mtl* mutants the amplified band is bigger in size due to the 44 base pair extension.

We analysed the genomic sequence of all coding exons of *Lmx1a* gene using primers specific for each exon (Table S1), covering the entire coding region and the splice sites. Then, exons were sequenced by capillary sequencing and traces were analysed. In *mtl* mutant mice no changes were found in the coding sequence of any exon of the *Lmx1a* gene. However, a single G to A base pair change was identified in the 3′ splice site of exon 4 (ENSMUSE00000223250) in transcript Lmx1a-001 (Fig. 3A–D), which is exon 5 (ENSMUSE00000223250) in transcript Lmx1a-002. This base pair transition c173+1 G>A found in *mtl* homozygotes abolishes the conserved GT in the AG/GT consensus rule, potentially preventing splicing at this position. Furthermore, an in-frame stop codon, TAA, is positioned three base pairs downstream of the base pair change (Fig. 3A–C). Exon 4 of transcript Lmx1a-001 and its flanking splice sites were sequenced in 17 other inbred strains (C57BL/6J, DBA/2J, A/J, 129S5, BXD-1/Ty, C58/J, CE/J, DDY/Jc1, FL/1Re, LP/J, NON/LtJ, RBG/Dn, St/bJ, SWR/J, C3HeB/FeJ, BALB/C, CBA/Ca) including the 129S5 strain that the original mutation is believed to have occurred in [19] and none of them showed this G to A transition identified in *mtl* homozygotes, which suggests that this change is not a common polymorphism and it can be used to genotype *mtl* mice.

PCR amplification of total cDNA from E10.5 embryos showed that in *mtl* homozygotes the amplified band encompassing exon 4 was larger compared to controls by approximately 50 base pairs (Fig. 3F). The sequencing of cDNA transcript from *mtl* mutants consistently showed a 44 base pair extension of exon 4 into intron 4, in contrast to wildtype transcripts (Fig. 3D). This, in turn, allows the in-frame stop codon located immediately after exon 4 to be read and to terminate the translation after 223 amino acids plus one altered amino acid instead of 382 amino acids (Fig. 3E, arrow). The newly activated cryptic splice site at the 3′ end of exon 4 in *mtl* homozygotes is the next available GTAT sequence (Fig. 3D, red lowercase letters) after 44 base pairs downstream of the wildtype 3′ splice site. In *mtl* mutants this mutation would result in an LMX1A protein, truncated in the homeodomain.

**Figure 4:**
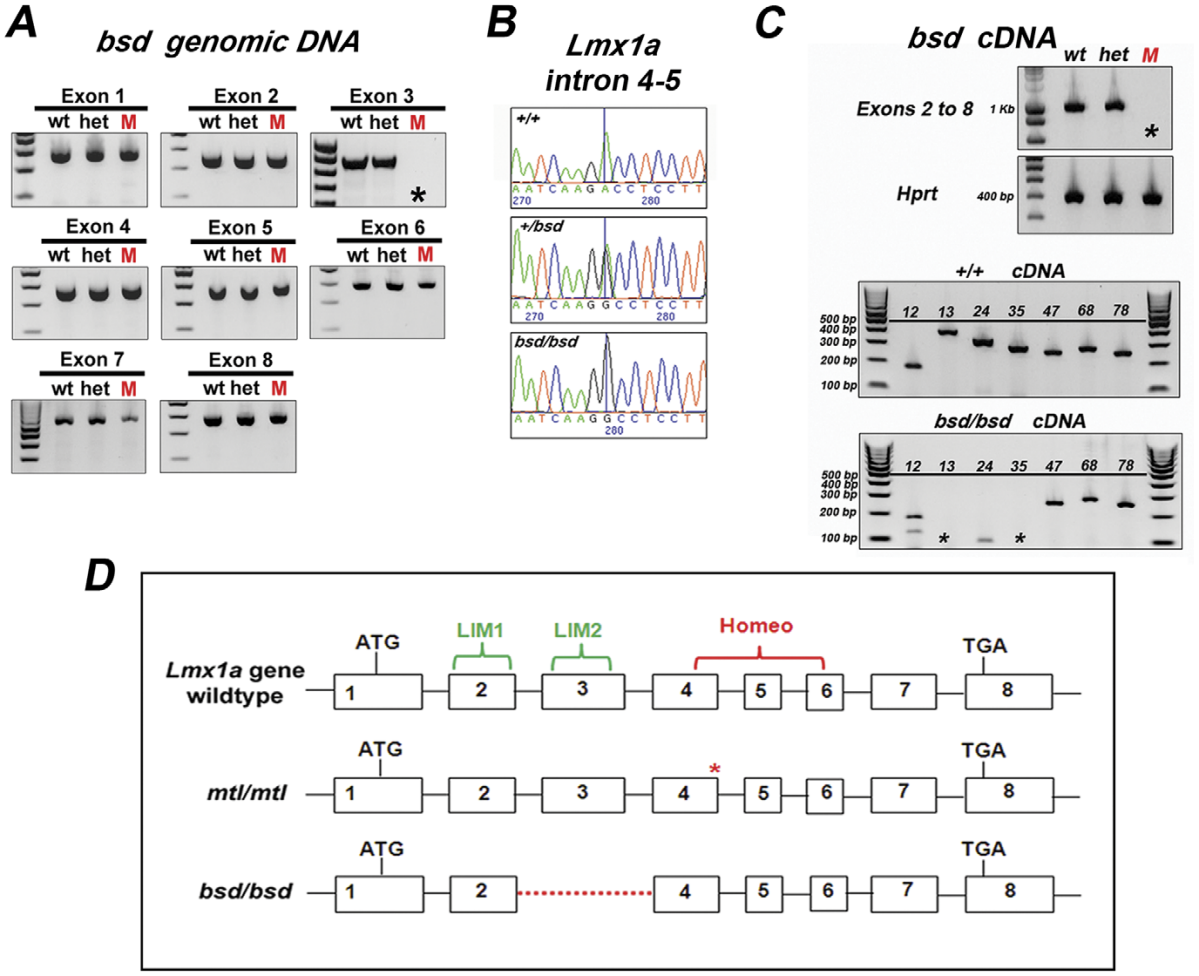
Deletion of exon 3 of *Lmx1a* in *bsd* mutants. A , PCR amplification of genomic DNA from +/+, +/*bsd* and *bsd*/*bsd* mice with primers specific to each exon of *Lmx1a* gene. One single band is amplified for each exon and sequenced for all *bsd* genotypes with no differences between mutants (M) and controls (wt, het) except for exon 3, where no band was amplified in *bsd* mutants (asterisk). **B**, Partial traces and sequence of *Lmx1a* intron 4–5 are shown. At 30 base pairs downstream of exon 4 we identified a single nucleotide polymorphism (SNP)-from A to G- that we used to genotype *bsd* mice. **C**, PCR amplification of *bsd* cDNA from +/+, +/*bsd* and *bsd*/*bsd* mice at E10.5 with primers specific to transcript Lmx1a-001 covering exons 2–8 (Table S2). Bands were amplified for controls (wt, het) but no band was detected in mutants (M). We designed primers for small fragments of the transcript (Table S2). Controls (+/+) showed bands for all combinations of primers (numbers on top indicate position of primers forward and reverse, respectively) whereas in *bsd/bsd* no bands were amplified by primers 13 and 35 (black asterisks). Interestingly, we found a small band of less than 100 bps with primers 24. This band is likely to correspond to the size of the amplicon (322 bp) minus the deleted exon 3 (233 bp) of *bsd* mutants. **D**, Diagram showing structure of *Lmx1a* gene in +/+, *mtl*/*mtl* and *bsd*/*bsd*. Initiation codon in exon 1 and termination codon in exon 8 are indicated. Point mutation in *mtl* homozygotes is indicated by a red asterisk and deletion in *bsd* mutants is shown by a red dotted line. *Mtl* mutation affects the homeodomain whereas in *bsd* the deletion of exon 3 involves the LIM2 domain.

In *bsd* mice we analysed the coding sequence plus flanking intronic regions of each of the *Lmx1a* exons by PCR and we amplified one single band for each exon, except for exon 3 (ENSMUSE00000223257 is exon 3 in transcript Lmx1a-001 and exon 4 in transcript Lmx1a-002) which gave no band (Fig. 4A, black asterisk). The analysis of the traces and sequences revealed that all exons were similar in wildtypes and *bsd* homozygotes except for exon 3, where no sequences were obtained for the mutants. In the analysis of the genomic DNA we found no variations in coding sequence of exon 4, however 30 base pairs downstream of the 3′ end of exon 4 there is a single nucleotide polymorphism (SNP) from A to G that segregates with the *bsd* phenotype (ENSMUSE00000223257 c173+34 A>G; position 1∶169,760,884 NCBIM37, Ensembl release 67) in its genetic background (Fig. 4B). This SNP is not unique but has not been identified in variants of the 129 genetic background previously (rs49997218 in SNPdb; Sanger Mouse Genome Project; [29]). This variation was used to genotype *bsd* mice. We also performed PCR amplification of the *Lmx1a* transcript in *bsd* cDNA at E10.5. Primers were located within exons 2 and 8, to encompass most of the transcript sequence. Our PCR results revealed that a single band was amplified for wildtype and heterozygotes (with no differences in the sequences) whereas no bands were amplified in *bsd* mutants (Fig. 4C). These results suggest that the *Lmx1a* mutation in *bsd* homozygotes might be preventing the amplification of the entire transcript. To further check whether part of the transcript could still be amplified, confirming that *bsd* mutation is within or around exon 3, we designed new primers to cover smaller regions of the *Lmx1a* transcripts. By PCR we amplified bands for exons 1 and 2, and 4 to 8 in all three *bsd* genotypes (+/+, +/*bsd*, *bsd/bsd*), however, the amplicon that should include exon 3 was missing (Fig. 4C, lines 13 and 35, asterisks) and we found a small band of less than 100 bp (around 90 bp) (Fig. 4C, line 24 in *bsd/bsd*) which might correspond to the amplicon 24 (322 bp) without exon 3 (233 bp). All these results are consistent with a deletion of exon 3.

To identify the breakpoints of the deletion we carried out PCR amplification on *bsd* genomic DNA of the regions flanking exon 3 and we found that the deletion comprises approximately 13.48 Kb (7.89 Kb upstream and 5.36 Kb downstream of *Lmx1a* exon 3) (Fig. S1 and Table S3). The consequences of translating *Lmx1a* mRNA without exon 3 (exon skipping) would result in truncation of LMX1A protein after 91 amino acids. Exon 3 is 233 bp long and it has a start phase of 2 and an end phase of 1 for its coding sequence. Thus, skipping this exon in the mRNA would result in a frameshifted reading for exon 4, resulting in turn in incorrect amino acids in positions 89 to 91 (FAV to QKR) and the termination of protein translation at a first stop codon in the position of amino acid 92.

In summary, the *mtl* mutation is a point mutation affecting the splicing of exon 4, which is part of the homeodomain, whereas the *bsd* mutation is a deletion of *Lmx1a* exon 3 which affects the coding of the LIM2 domain (Fig. 4D).

### The *mtl* and *bsd* Mutations are most Likely Derived from ES Cells Used for Transgenesis

The *mtl* mutation arose as a by-product of an experiment initially aimed at replacing, by homologous recombination, the homeogene Hox-3.1(Hoxc-8) by the E.coli lacZ gene [19], [30]. Among the ES cell clones obtained and used to generate germ-line chimeric males, one of them turned out to be a false positive. A mouse strain was obtained in which the pGN targeting vector [19] was inserted in a undetermined genomic location. As the pGN plasmid carried the Neo^R^ resistance cassette the mouse strain was kept to produce Neo-resistant embryonic fibroblast. To facilitate this production, homozygous males and females for the pGN insertion were generated and then hemizygous sisters and brothers were intercrossed. One of the F1 litters gave one male with circling behaviour. The analysis of this phenotype over several generations showed that the mutation was a recessive mutation not linked to the pGN insertion (Yvan Lallemand, personal communication).

Homozygous animals showing the *bsd* phenotype were first encountered in the F2 generation of 2 newly established transgenic colonies created for complementation of a different gene. Both colonies arose from independent chimaeric founder males carrying the same transgenic construct, but were independent ES cell clones after using the recombination mediated cassette exchange (RCME) approach and antibiotics selection. Thus, we checked at which stage of genetic manipulation the mutation most likely arose.

The SNP we now use for genotyping *bsd* can be traced through all stages of transgenesis starting with CCI 18 ES cells DNA [31]. These are male 129S5/SvEvBrd-*Hprt*_AB1 ES cell clones carrying plasmid CCI18. The SNP is present in heterozygosis in those ES cell clones at all stages prior and post transgenic modifications before blastocyst injection (Fig. S2). The two independent chimeric colony founder males used to establish transgenic colonies were heterozygous for the genotyping SNP as well. However, PCR of the ES cell clones for the genomic region of *Lmx1a* exon 3 and in intron 2–3 gives rise to a PCR product of wildtype length (data not shown) so it doesn’t uncover a deletion, presumably because it is present in heterozygous form. In both mouse colonies we found a linkage of the SNP and the *bsd* phenotype. The appearance of *bsd* in two independent colonies derived from the same ES cells suggests a shared origin, which can only have arisen in the ES cell colony prior to microinjection but after genetic manipulation of the ES cells.

### Morphological Defects in the Inner Ears of *mtl* and *bsd* Mutants

**Figure 5:**
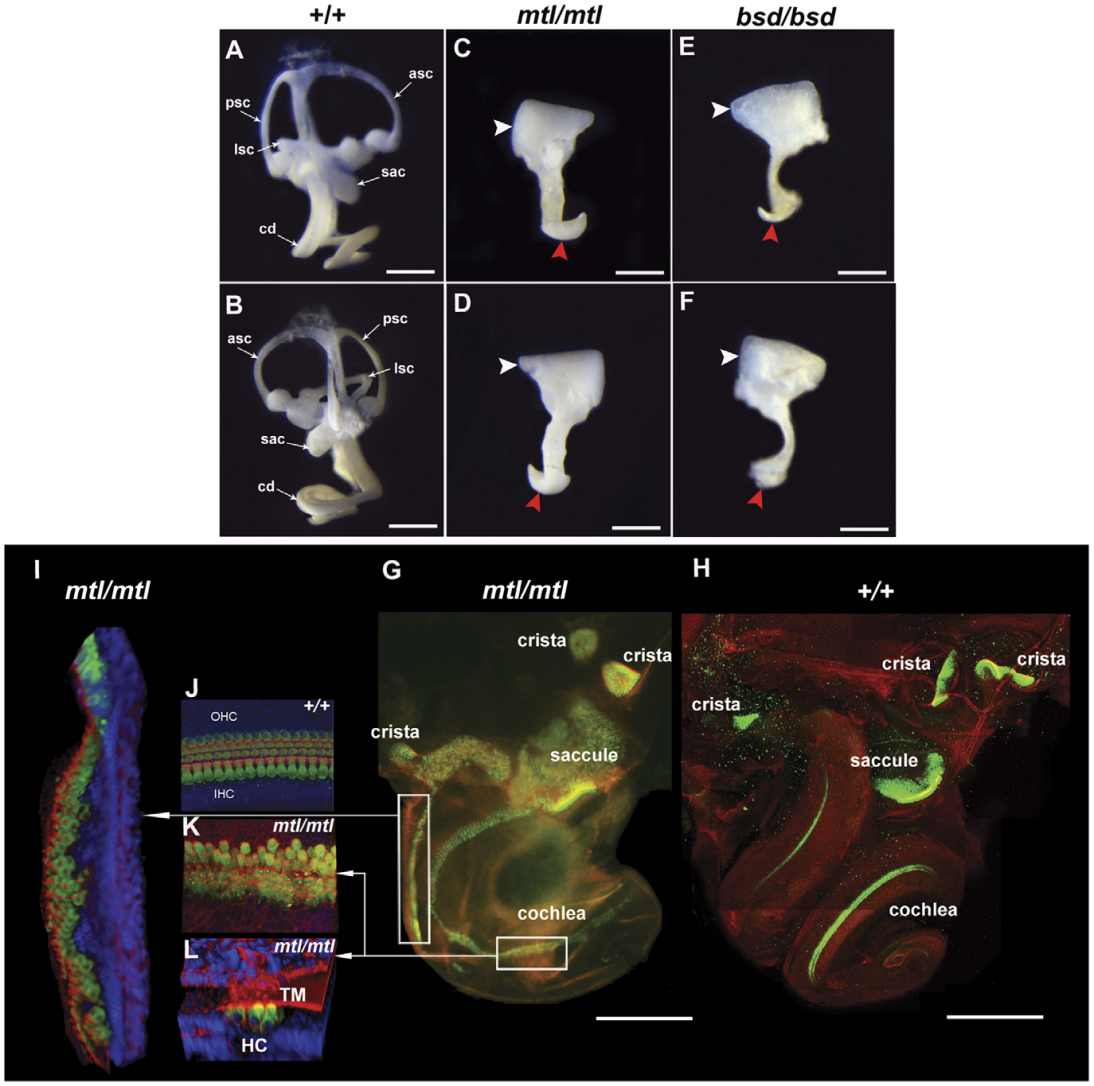
Morphology of inner ears and analysis of sensory patches in mutants. **A–F**, Paint-filled inner ears from *mtl* and *bsd* mutants at E16.5. Inner ears (front and back views) from wildtype (**A, B**), *mtl*/*mtl* (**C, D**) and *bsd*/*bsd* (**E,** **F**) mice are shown. Both homozygotes show under-developed cochlear ducts (red arrowheads) compared to their wildtype littermates. Vestibular system in homozygotes appears like a cyst without semicircular canals (white arrowheads). **G**, **H**, Confocal imaging and 3D reconstruction of P0 inner ears of one *mtl* mutant (**G**) and one wildtype (**H**). We used Myo7a (green) for sensory hair cells, rhodamine-phalloidin (red) for actin and DAPI (blue) for cell nuclei. The expression of Myo7a shows the main sensory patches in the inner ear (anterior, posterior and lateral cristae, utricle and saccule in vestibular system and organ of Corti in the cochlea) (**H**). In *mtl* mutants cristae can be identified by their relative position in the otocyst and utricle and saccule domains look abnormal compared to those in wildtypes (**G,** **H**). In *mtl* mutants there are two continuous Myo7a-positive hair cell populations descending to the truncated cochlea (insets). One of these populations show a quite uniform organization of the hair cells within the organ of Corti but without a tectorial membrane (**I**, area boxed in **G**), whereas the other population extending to a more distal location within the cochlea (**K**, **L**, area boxed in **G**) displays properties of a developing organ of Corti containing hair cells and a tectorial membrane (**L**). (**K** and **L** are reconstructions from the same confocal image stack. **K**, Organ of Corti surface, image sub-stack underneath tectorial membrane; **L** is a rotated and tilted 3D reconstruction of the same image stack). Asc, anterior semicircular canal; cd, cochlear duct; ed, endolymphatic duct; lsc, lateral semicircular canal; psc, posterior semicircular canal; sac, saccule, OHC, outer hair cells; IHC, inner hair cells; TM, tectorial membrane. Scale bars: **A–F**, 200 µm; **G–H**, 500 µm.

We examined inner ears by clearing with glycerol and we found that in *bsd* homozygotes inner ears were smaller than in controls, with severely malformed cochlear ducts and vestibular systems (data not shown). To investigate the morphological defects in embryonic stages we performed paint-filling analysis of *mtl* and *bsd* inner ears at embryonic day 16.5 (E16.5). The inner ears of both *mtl* and *bsd* mutants looked severely malformed, with no semicircular canals in the vestibular system and with short cochlear ducts. The entire vestibular system was replaced by a cyst-like structure and the mutant ears also lacked an endolymphatic duct (Fig. 5A–F).

### Homozygous *mtl* Mice Show Severe Defects in Sensory Patch Arrangement

To further analyse the morphogenesis of the sensory patches within the inner ear we examined the organization of the sensory patches in whole mount preparations of newborn mice (P0). There are six distinct sensory organs in the mammalian inner ear: the cochlea contains the organ of Corti and the vestibular system contains anterior, lateral and posterior cristae, macula of the utricle and macula of the saccule. At P0 the cochlear duct has extended to its full length and the vestibular system with the semicircular canals is already formed. Based on the expression of protein Myosin7a (Myo7a) (Fig. 5H) the six major mammalian sensory patches can be identified in a whole mount preparation where the surrounding bone capsule has been removed (Fig. 5H- utricle is not visible since it is concealed by the lateral semicircular canal and basal hook of the cochlea). In *mtl* mutants, the inner ear appears as an enlarged otocyst with a single continuous lumen (Fig. 5C, D, G). Dissected epithelium lining the inside of the bone capsule shows a short curved extension, identifiable as a truncated and malformed cochlear duct (Fig. 5G).

In *mtl* mutants we identified the three cristae based on the conserved relative position in the otocyst (Fig. 5G, H). The malformed utriculo-saccular domain populates the area next to a zone where the epithelium is narrowing to form the cochlear duct. We observed two continuous hair cell populations expressing Myo7a descending to the cochlear duct (Fig. 5G, insets). The population more distally located within the cochlea displays outer hair cells (OHC) and inner hair cells (IHC) judged by their position and distribution along the truncated cochlear duct and their association with a tectorial membrane (Fig. 5K, L). Inner hair cells show a more uneven organisation than in wildtypes and can form more than a single row; this uneven organisation has been described also in other *Lmx1a* alleles [13], [14]. The other population of sensory hair cells descending from the posterior pole of the otocyst (vertical box in Fig. 5G) and towards the basal turn of the cochlea is not associated with an obvious tectorial membrane (Fig. 5I). This extra strip of sensory hair cells in the basal turn of the cochlea has been reported previously [14]. In addition, the postnatal inner ear in *mtl* homozygous mice showed no indications of otoconia associated with the vestibular maculae. The bone capsule and the mutant inner ear of older mice looked free of pigmented areas. Finally, we found no evidence of expansion of the areas populated by Myo7a-positive cells between P0 and P7 in *mtl* mutants (data not shown).

We also analysed the arrangement of sensory patches in sections, using markers specific for sensory and non-sensory tissue within the cochlea and vestibular system of *mtl* mutants. We followed the expression of Sox2, Jag1 and p27^Kip1^ (early markers of the prosensory domains) through development and found that at E14.5 vestibular and cochlear sensory patches are present in the otocyst of *mtl* mutants (data not shown). Cristae are identifiable by a circular or ellipsoid shape, their relative position to each other in the otocyst and spaced by large areas of epithelium negative for Sox2 and Jag1 surrounding them. The maculae of the utricle and saccule in the mutants do show clearly expression of Sox2 and Jag1; however the patches are poorly-defined compared to wildtype utricular and saccular sensory patches. The mutant maculae were also detected in the vestibular pole of the otocyst, but much closer to the abnormal cochlear duct. In mutants we also found expression of Sox2, Jag1 and p27^kip1^ markers in the rudimentary and partially extended cochlear duct (data not shown).

**Figure 6:**
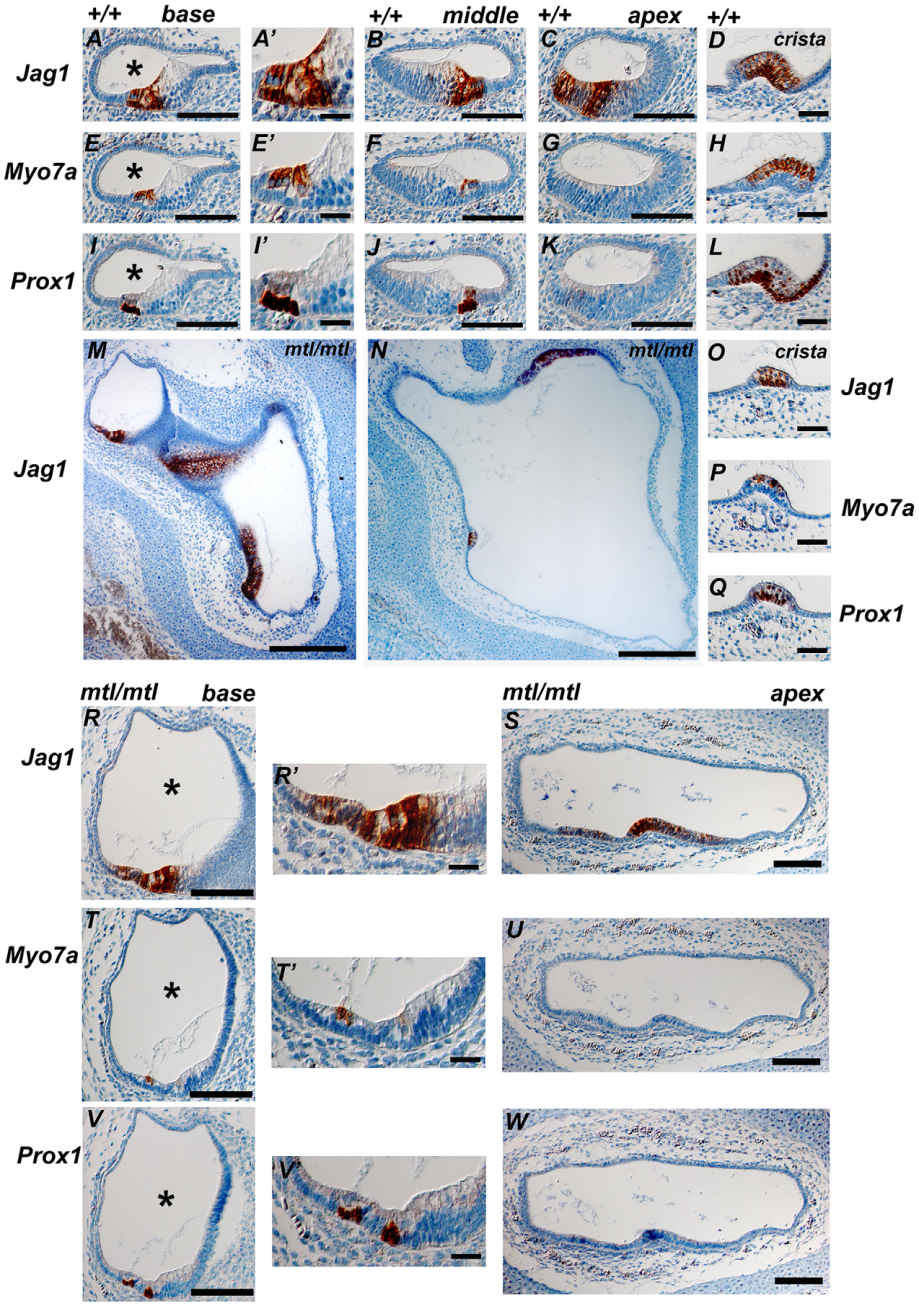
Analysis of sensory patch formation in cochlear and vestibular sections of *mtl* mutants at E16.5. Expression of markers for sensory patch development, Jag1 (**A, A’, B–D, M–O, R, R’, S**), Myo7a (**E, E’, F–H, P, T, T’, U**) and Prox1 (**I, I’, J–L, Q, V, V’, W**) are shown (**A’, E’,I’, R’, T’** and **W’** are high magnifications of **A, E, I, R, T** and **W**, respectively). (**A–L, A’, E’, I’**) are wildtypes at E16.5. (**M–W, R’, T’, V’**) are *mtl/mtl* at E16.5. (**D, H, L**) Posterior crista (PC) is positive for these markers at E16.5. In *mtl* mutants, vestibular labyrinth formation fails and, instead, a large cystic otocyst with a wide cochlear duct can be observed (**M, N**), with enlarged endolymphatic space (**R–W**). Sensory patches are positive for Jag1 and the expression is detected in *mtl* mutants although in abnormally extended pattern. The mutant cristae (**O–Q**) show expression of the three markers and a similar degree of organization (multilayered, bulged epithelium, layered expression of Myo7a and Prox1) as in wildtypes. The utrico-saccular sensory patches in *mtl* mutants are disorganized but express the sensory patch markers. In the base of the mutant cochlear duct (**R, T, V**) all three markers are expressed, but the pattern is aberrant (**R’, T’, W’**) labelling two sensory patches compared to controls where the single organ of Corti is labelled. The apex of the truncated mutant cochlear duct (**S, U, W**) shows Jag1 expression, faint Prox1 expression and no Myo7a expression, reflecting the baso-apical differences in hair cell development of the littermate controls. Asterisks show the endolymphatic compartment, which is extremely enlarged in *mtl* mutants (R, T, V) compared to wildtypes (A, E, I). Scale bars: **A, E, I, B, F, J, C, G, K, M, N, R–W,** 200 µm; **A’, E’, I’, D, H, L, R’, T’, V’**, 50 µm.

At E16.5 the mutant otocyst has continued to grow and the sensory patches can be identified based on the presence of Jag1 (expressed in the prosensory domain), Myo7a (early marker of post-mitotic sensory hair cells) and Prox1 (predominantly expressed in supporting cells). Jag1 expression can be detected throughout the length of the cochlear duct of control littermates (Fig. 6A–C). Myo7a expression can be detected at this stage at the basal and middle turns of the cochlea but it is not detected at the apex (Fig. 6E–G). Finally, Prox1 expression was found in the supporting cells of the cochlea at the basal and middle turns but not in the apex (Fig. 6I–K). In the mutants, the basal turn of the cochlea expresses all three markers, although their expression patterns looked abnormal (Fig. 6R, T, V). The apex of the truncated mutant cochlear duct expresses Jag1 and a weak staining of Prox1, whereas there is no expression of Myo7a detected (Fig. 6S, U, W), reflecting the base-to-apex differences in the hair cell development of the control littermates. We also found a more extended expression of Jag1 in apical sections of the mutant cochlea. Finally, an extremely enlarged endolymphatic compartment was also evident in the mutant cochlea (Fig. 6, asterisk, compare wildtypes A, E and I with *mtl* mutants R, T and V).

In the vestibular system, the posterior crista (PC) expresses these three markers at E16.5 (Fig. 6D, H, L). In *mtl* mutants, vestibular labyrinth formation fails and instead a large cystic otocyst with a wider cochlear duct can be observed (Fig. 6M, N). There are sensory patches present in this structure (Fig. 6M, N) as identified by Jag1 expression. The mutant cristae show expression of the three sensory patch markers and a similar degree of organization (multilayered, bulged epithelium and layered expression of Myo7a and Proxi1) compared to wildtypes (Fig. 6O, P, Q). Similarly to wildtypes, the utriculo-saccular sensory patches in the mutant also express Myo7a at the surface and Prox1 below.

### Expression Analysis of *Lmx1a*, FOXI1 and FGF9 in the Developing Inner Ear of *mtl* Mutants

**Figure 7:**
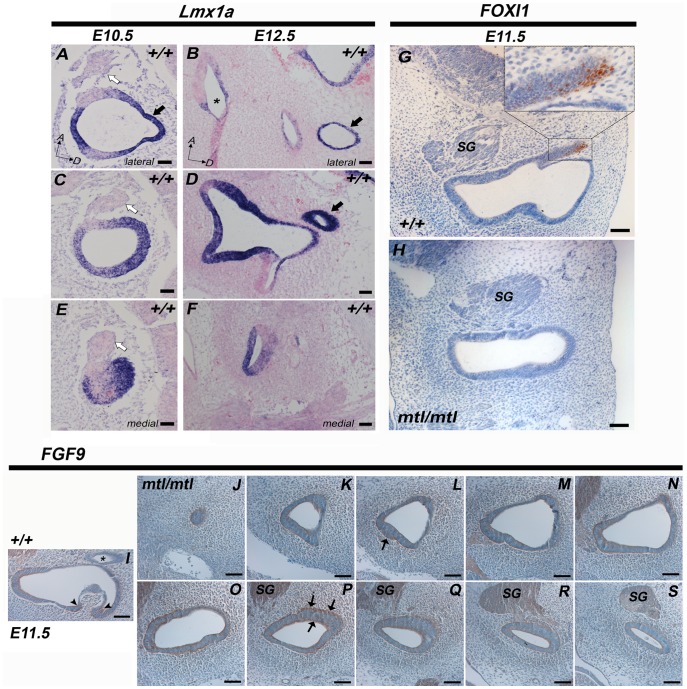
Expression analysis of *Lmx1a*, FOXI1 and FGF9 in the developing inner ear. A–F , *In situ* hybridization of *Lmx1a* on wildtype inner ear sections at E10.5 (**A, C, E**) and E12.5 (**B,** **D, F**). *Lmx1a* mRNA expression is shown in blue and nuclear counterstain in pink. In wildtype inner ears *Lmx1a* is expressed in the otic vesicle and in the endolymphatic sac (black arrow in **A,** **B** and **D**), whereas delaminating neuroblasts are *Lmx1a-negative* (**A,** **C,** **E,** white arrows). At E12.5 *Lmx1a* expression is also found in the region of the fusion plates which will later form the semicircular canals (asterisk in **B**). *Lmx1a* mRNA expression appears to be restricted to prospective non-sensory inner ear tissue. **G–H**, In wildtypes FOXI1 is specifically expressed in a subpopulation of cells within the endolymphatic sac (**G**) whereas in *mtl* mutants the typical evagination of the endolymphatic sac/duct fails and no FOXI1-positive cells are detected in or adjacent to the otic vesicle/otocyst (**H**). **I–S**, At E11.5 FGF9 expression highlights areas of epithelial thickening on the luminal as well as the mesenchymal side of the otic vesicle epithelium, such as the outpocketings of the developing semicircular canals (arrows in **I**). The endolymphatic duct is negative for FGF9 (asterisk in **I**). **J–S**, Series of sections covering the complete otocyst arranged from lateral to medial, of one *mtl* homozygote showing only a rudimentary otocyst epithelium rearrangement. Sections also show complete absence of an endolymphatic duct/sac structure. FGF9 is expressed in the mutant epithelium and marks epithelial thickening, but unlike in wildtypes there is no semicircular canal formation. The luminal and mesenchyme sides of the thickened mutant epithelium looked underdeveloped (arrows in **I** and **P**). SG, spiral ganglion. Scale bars: 100 µm.

We analysed *Lmx1a* expression by *in situ* hybridization in sections at E10.5 and E12.5. In E10.5 wildtypes *Lmx1a* is widely expressed in the otic vesicle. Neuroblasts delaminating from the otic vesicle are *Lmx1a*-negative (Fig. 7A, C, E, white arrows). *Lmx1a* is highly expressed in the endolymphatic sac/duct throughout the stages of its morphogenesis (Fig. 7A, B, D, black arrows). There is also high expression in the fusion plates at E12.5 (Fig. 7B, asterisk) which later will fuse to form the semicircular canals. In tissues forming the labyrinth and the cochlear duct, *Lmx1a* mRNA is restricted to prospective non-sensory tissue.

The inner ear of embryonic and newborn *mtl* mutants appeared like a cyst structure with only a single continuous lumen. This abnormal morphology was first observed at E10.5, when in the wildtype endolymphatic duct/sac (ED/ES) formation begins. The endolymphatic duct formation seems to be the first morphogenetic event in the inner ear development after formation of otic cup and otic vesicle, and *Lmx1a* mRNA is expressed during the entire period critical for morphogenesis of the ED/ES system. We used FOXI1 as an early molecular marker of the ED/ES epithelium. FOXI1 is expressed in the entire otic vesicle at E9.5 and it becomes gradually restricted to a subpopulation of cells within the ED/ES epithelium [32] and in *Foxi1* ^−/−^ mice an abnormal expansion of the endolymphatic compartment is detected [32]. In wildtype embryos at E11.5 we found expression of FOXI1 within the ED/ES epithelium (Fig. 7G) whereas in *mtl* mutants no expression of FOXI1 was detected and the morphology of the otic vesicle looked abnormal with no ED/ES development (Fig. 7H). The analysis of sections through the entire embryo gave no indications of ectopic expression of FOXI1 within the otic vesicle or surrounding tissues. Wildtype sections show a normal epithelial thinning adjacent to the site of ED/ES formation (Fig. 7G), whereas in the mutants the otocyst shows only very rudimentary changes in epithelium thickness where the ED/ES should arise.

We also analysed the expression of FGF9 (Fibroblast growth factor 9) as an early marker of semicircular canal formation [33]. In *Fgf9* ^−/−^ mice there are no semicircular canals and instead a large cavity is observed [33], similar to that of *mtl* and *bsd* homozygous mice. In wildtype mice at E11.5 FGF9 is detected at the edges of the outpocketings of the developing semicircular canals and coincides with areas of epithelial thickening in wildtype mice (Fig. 7I, arrowheads). In mutants the outpocketing process fails, FGF9 expression in the epithelium looked diffuse, and the otocyst does not undergo morphogenesis to give rise to semicircular canals. The epithelium in the mutant otocyst appears irregular and at sites of epithelial thickening wavy and uneven (Fig. 7J–S).

### *Lmx1a* is Down-regulated in *mtl* and *bsd* Mice and so are *Hmx3, Hmx2, Netrin1* and *Pax2*

**Figure 8:**
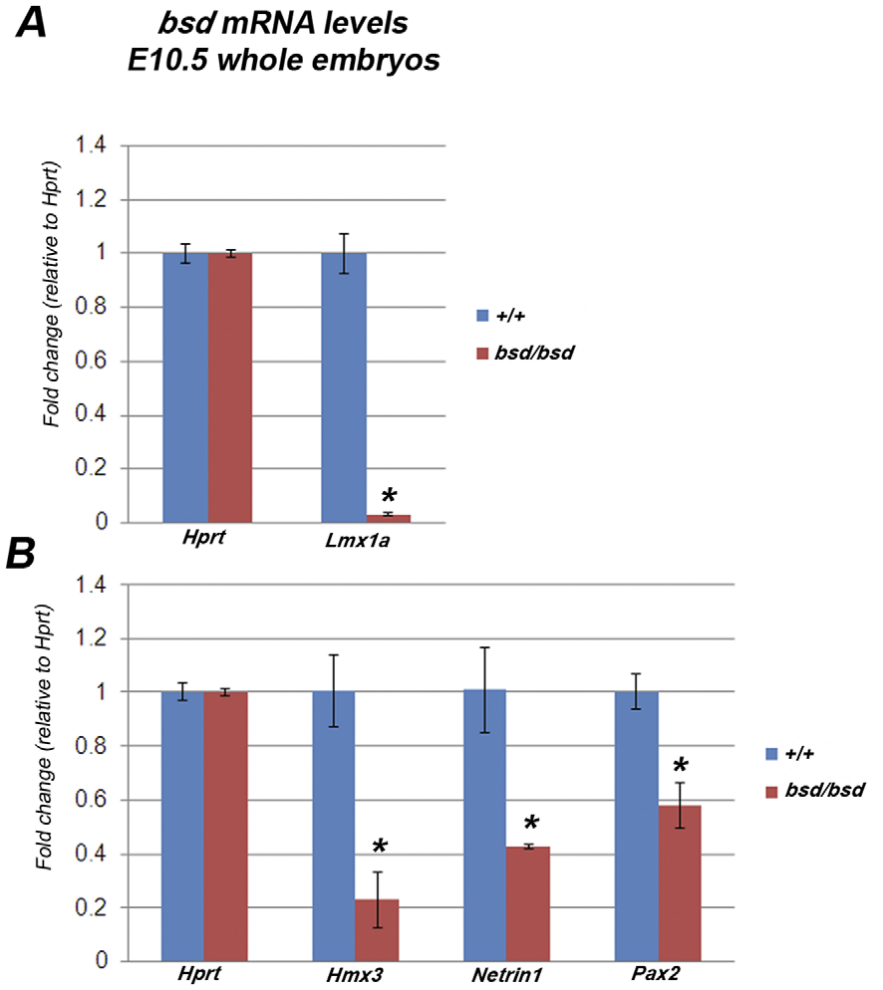
Expression analysis of *Lmx1a*, *Hmx3*, *Netrin1* and *Pax2* mRNA levels in *bsd* mutants. Quantitative real-time PCR on cDNA generated from normalized total RNA from *bsd* E10.5 whole embryos (only *bsd* results are shown). **A**, *Lmx1a* is significantly down-regulated in *bsd* homozygous mice at E10.5 (n = 5+/+, n = 5 bsd/bsd; *P<0.05, *t test*; *mtl* data not shown). **B**, *Hmx3*, *Netrin1* and *Pax2* are also significantly down-regulated in *bsd* mutants (n = 5+/+, n = 5 bsd/bsd; *P<0.05, *t test*; *mtl* data not shown). Error bars represent standard deviations. Quantities normalized to *Hprt* levels. *Hprt* was used as control for the quantity of material.

The quantitative analysis of *Lmx1a* mRNA levels in *mtl* and *bsd* mutants clearly showed a significant down-regulation (n = 10*+/+*, n = 5 *mtl/mtl*, n = 5 *bsd/bsd*; *P*<0.05, *t* test) in whole embryos at E10.5 (Fig. 8A, only *bsd* results are shown). Following these results we asked if these mutations in the *Lmx1a* gene might affect the expression of other genes considered critical for normal early morphogenesis of the vestibular system and cochlea along the dorsolateral to ventromedial axis. For instance, above we have described the total loss of FOXI1 positive cells at the dorsolateral pole of the prospective inner ear and the associated lack of any endolymphatic duct/sac observed in *mtl* and *bsd* mutants. Similarly, we especially looked at genes which are expressed in the otic vesicle at E10.5 in patches that overlap with the expression domain of *Lmx1a*. We checked the mRNA levels and protein expression of those genes or transcription factors which, when mutated, show a phenotype that resembles, at least partially, the features of the phenotype shown by *bsd* and *mtl* mutants.

**Figure 9:**
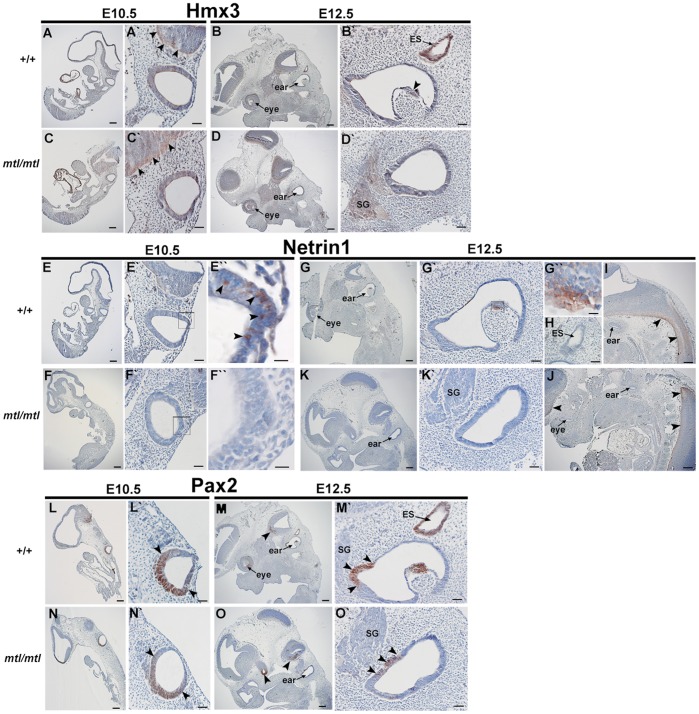
Expression analysis of Hmx3, Netrin1 and Pax2 by immunohistochemistry. Expression of Hmx3 protein was performed on wildtype (**A, A’, B, B’**) and *mtl* mutant sections (**C, C’, D, D’**) at E10.5 (**A, A’, C, C’**) and E12.5 (**B, B’, D, D’**). At E10.5 Hmx3 protein expression in the otocyst looks diffused in wildtypes and mutants (**A’, C’**) and expression in CNS is also strong (**A’, C’**, arrowheads). At E12.5 there is strong expression of Hmx3 in the ED/ES (**B’**) and in the outpocketings of the developing semicircular canals (**B, B’**, arrowhead). In *mtl* mutants no semicircular canals or ED/ES are found and a simple cyst is observed instead (**D, D’**). Netrin1 protein expression was analysed in wildtype (**E, E’, E’’, G, G’, G’’,H, I**) and *mtl* mutants (**F, F’,F’’,K,K’,J**) at E10.5 (**E, E’, E’’, F, F’, F’’**) and E12.5 (**G,G’,G’’,H,I,J, K,K’**). Netrin1 protein was detected in the dorsolateral margin of the wildtype otocyst (**E’, E’’**) whereas mutants did not show any expression (**F, F’’**). In wildtypes at E12.5 strong expression of Netrin1 protein is found in the outpocketings of the developing semicircular canals (**G’**, magnified view in **G’’**) but no expression was found in the ED/ES (**H**). Similar expression was detected in the neural tube of wildtypes and *mtl* mutants (**I, J**, arrowheads). Pax2 protein expression was analysed in wildtype (**L, L’, M, M’**) and *mtl* mutants (**N, N’,O, O’**) at E10.5 (**L, L’, N, N’**) and E12.5 (**M, M’, O, O’**). At E10.5 Pax2 protein expression is observed in the ventral region of the otocyst in wildtypes and mutants (**L’, N’**). In wildtypes at E12.5 strong signal is found in ED/ES, in outpocketings of the semicircular canals and in a ventral margin of the otic vesicle (**M’**, arrowheads). In *mtl* mutants the ED/ES and semicircular canals are missing and no expression can be found except for the marginal region of the presumptive cochlear duct, which still shows Pax2 expression (**O’**, arrowheads). Scale bars: (**A–G, I–O**), 200 µm; (**A’–G’, H, K’, L’–O’**) 50 µm; (**E’’–G’’**) 10 µm.

Previous reports have shown that the transcription factor *Hmx3* is one of the earliest markers for the development of the vestibular system and it plays a key role in semicircular canal formation [34], [35], [36]. At E10.5 *Hmx3* is detected in the otic vesicle, the dorsolateral region of the neural tube, dorsal root ganglia and optic cups [34]. *Hmx3* knockouts show severe vestibular defects with a fusion of the utricle and saccule into a single utriculo-saccular cavity [35], similar to *mtl* mutant mice. *Hmx2* is a transcription factor closely related to *Hmx3*, which has a critical role in vestibular morphogenesis as well [37]. *Hmx2* is detected at E9.5 in the anteriodorsal portion of the otic vesicle and from E12 is strongly expressed in the CNS, including developing neural tube, pons and hypothalamus [37]. *Hmx2* knockouts show severe vestibular malformations and complete lack of semicircular canals [37], [38]. However, in contrast to *mtl* and *bsd* mutants, cochlear morphology is not grossly affected in *Hmx2^−/−^* and *Hmx3^−/−^* mice. We found that in both *bsd* and *mtl* mutants there is a significant down-regulation of *Hmx3* mRNA transcripts (n = 10+/+; n = 5 *mtl/mtl*, n = 5 *bsd/bsd*; *P*<0.05, *t* test) in whole embryos at E10.5 (Fig. 8B, only *bsd* results are shown). However, immunohistochemistry of sections of E10.5 *mtl* mutant embryos showed a similar level of diffuse expression of Hmx3 in mutant otocysts compared with wildtypes (Fig. 9A, A’, C, C’); the reduced mRNA levels in whole embryos may have resulted from downregulation elsewhere in the embryo. *Hmx2* mRNA levels were also significantly reduced in whole embryos at E12.5 (when *Hmx2* starts to be detected; data not shown). In E12.5 wildtypes, Hmx3 protein was detected in the CNS and strong expression was found in ED/ES (Fig. 9B, B’) and the outpocketings of the developing semicircular canals (Fig. 9B’ arrowhead). In *mtl* mutants the outpocketing process fails and no ED/ES is formed but Hmx3 protein still looked diffuse in the inner ear and strong in CNS (Fig. 9D, D’).

*Netrin1* (*Ntn1*) is a member of the laminin-related secreted proteins required for the normal morphogenesis of the semicircular canals. At E10.5 it is expressed in the dorsolateral wall of the otic vesicle which will form the fusion plate leading later to the formation of the semicircular canals [39]. *Netrin1* knockout mice have a reduced anterior semicircular canal and complete lack of the posterior and lateral canals, although *Netrin1* mutation does not severely affect the cochlear morphogenesis [39]. In *mtl* and *bsd* mutants there is a complete absence of semicircular canals and we found that *Netrin1* mRNA levels are significantly down-regulated (n = 10+/+; n = 5 *mtl/mtl*; n = 5 *bsd/bsd*; *P*<0.05, *t* test) in whole embryos at E10.5 (Fig. 8B). Expression of Netrin1 protein in neural tube and otic vesicle was detected in wildtype embryos at E10.5 in the dorsolateral wall of the otocyst (Fig. 9E, E’, E’’), but in *mtl* mutants no expression was detected in otic vesicles (Fig. 9F, F’, F’’). In wildtypes at E12.5, Netrin1 is clearly detected in the outpocketings of the developing semicircular canals (Fig. 9G, G’, G’’) but this structure fails to form in *mtl* mutants and a simple cyst is found instead (Fig. 9K, K’). Netrin1 was not detected in ED/ES of wildtypes (Fig. 9H). Expression of Netrin1 protein in neural tube remains apparently unaffected in mutants compared to controls (Fig. 9I, J, arrowheads).

*Pax2* is a paired box transcription factor which is expressed in the medial region of the mammalian otocyst at E10.5, as well as in the endolymphatic duct and other non-sensory regions [40]. Mutations in *Pax2* cause complete agenesis or severe malformation of the cochlea and the spiral ganglion but normal development of the vestibular system [41]. The endolymphatic sac in these mutants is present although malformed [42]. In *mtl* and *bsd* homozygotes we observed a short and malformed cochlear duct which resembles that shown by *Pax2* ^−/−^ mice and our RT-PCR analysis showed that *Pax2* mRNA levels are significantly down-regulated (n = 10+/+; n = 5 *mtl/mtl*; n = 5 *bsd/bsd*; *P*<0.05, *t* test) in whole mutant embryos at E10.5 (Fig. 8B). We found strong expression of Pax2 protein in the ventromedial region of wildtype otocysts at E10.5 and it was also strongly expressed in brain and eye (Fig. 9L, M, N, O). A similar expression pattern was found in otocysts of E10.5 *mtl* mutants (Fig. 9L, L’, N, N’, expression domain between arrowheads), suggesting that the downregulation of *Pax2* in whole mutant embryos at this stage may be due to reduced mRNA levels elsewhere in the body. In E12.5 wildtypes a strong Pax2 protein expression was found in the outpocketings of the developing semicircular canals, ED/ES and in a medial region of the presumptive cochlea that will develop into the lateral wall of the cochlear duct (Fig. 9M, M’, O, O’, arrowheads). In *mtl* mutants, however, expression of Pax2 protein was observed only in the presumptive cochlea, since the prospective tissue that will develop into semicircular canals and ED/ES fails to form (Fig. 9O’, arrowheads). Expression in brain and eye was apparently unaffected in *mtl* mutants compared to controls (Fig. 9M, O).

Taken together, our results suggest that Hmx3 and Pax2 are expressed in the *mtl* mutant otocyst at E10.5 and are not directly affected by absence of *Lmx1a*, although they are not detected in the ear at E12.5 following abnormal morphogenesis. However, Netrin1 expression is absent in the otocyst of *mtl* mutants at E10.5, suggesting Lmx1a acts upstream of *Ntn1*.

## Discussion

### *Mtl* and *bsd* are Two New Mutant Alleles of *Lmx1a* Gene

Here we present *mtl* and *bsd*, two novel mutant alleles of the *Lmx1a* gene. The similarities in the phenotype between these two mutants and the previously described *shaker-short* [2] and *dreher* mutations (*Lmx1a^dr^*) [3], [15], [16], [17], [18] and *Lmx1a^dr-J^*; [4], [13], [14] along with the non-complementation of these mutations clearly pointed to *Lmx1a* as the gene involved. Homozygous *mtl* and *bsd* mice are smaller, show circling and head-bobbing behaviours, display white belly patches, have short/blunt tails, and are profoundly deaf. Our further analysis revealed severe defects in the inner ear morphology including a malformed vestibular system with a cyst-structure without semicircular canals or endolymphatic duct, and a truncated cochlear duct.

Our molecular analysis revealed that in *mtl* mutants there is a G to A transition affecting the 3′ splice site of exon 4 causing the truncation of the LMX1A protein, whereas in *bsd* mutant mice we found a deletion of exon 3 plus part of flanking intronic regions causing the non-amplification of the *Lmx1a* transcript. For *bsd* we conclude that the deletion arose in a male 129S5/SvEvBrd-*Hprt*_AB1 derived ES cell clone. In these ancestral ES cells we failed to find a deletion by PCR, but since it is likely that the deletion was present on one chromosome only then standard PCR would not be able to uncover this from genomic DNA.

Our complementation data and the Mendelian inheritance with fully penetrant phenotype in homozygotes together with the nature of the mutations we detected strongly suggest the classification of *mtl* and *bsd* mutants as recessive null alleles of *Lmx1a*.

### Placing *mtl* and *bsd* Mutants within the *Lmx1a* Allelic Series

Chizhikov et al [5] reported an allelic series of *Lmx1a* mutations placing five of eleven molecularly defined mutant alleles (*Lmx1a^dr-sst^, Lmx1a^dr-3J^, Lmx1a^dr-7J^, Lmx1a^dr-2J^, Lmx1a^dr-6J^*) as protein null alleles based on sequence analysis, because they either carry deletions of the ATG translation initiation codon (*Lmx1a^dr-sst^, Lmx1a^dr-3J^, Lmx1a^dr-7J^* ), the transcription termination TGA codon and polyadenylation stop signal (*Lmx1a^dr-6J^*) or cause a frameshift with very early termination of the protein (*Lmx1a^dr-2J^*). In contrast, *Lmx1a^dr-J^*, carrying a Cys82Tyr mutation, along with two other *Lmx1a* alleles (*Lmx1a^dr^* and *Lmx1a^dr-Kjmi^*), are predicted to generate an altered protein. Koo et al [14] suggest that *Lmx1a^dr-J^*, which is the most widely analysed allele, is likely to be a functional null because the single G to A point mutation alters a conserved cysteine essential for LIM domain function. For *mtl* and *bsd* mutants we failed to amplify the *Lmx1a* transcript by PCR of cDNA and the quantification of the mRNA levels of *Lmx1a* transcript by RT-PCR revealed a significant down-regulation. This down-regulation may be exacerbated by the auto-regulatory function of *Lmx1a in vitro*, demonstrated by Chung et al [43]. Our results suggest that both new mutations in *Lmx1a* might be preventing the production of a functional protein which results in two novel null alleles of *Lmx1a*.

Among the described alleles, the high abundance of genomic deletions within the Lmx1a locus (*Lmx1a^dr-4J^*, *Lmx1a^dr-7J^, Lmx1a^dr-8J^, Lmx1a^bsd^)* is striking. A systematic bioinformatic analysis of the genomic area of the *Lmx1a* locus for facilitators of such genomic deletions (e.g. transposons) could address the question of whether the locus itself is more prone to small genomic deletions than others.

### Inner Ear Morphogenesis is Severely Affected in *mtl* and *bsd* Mutants

All phenotypes that segregate with *mtl* and *bsd* homozygosis are fully penetrant although subject to a limited variability. This variation is larger for the white belly spot/belt and tail length than for the gross morphology of the mutant otocyst at early postnatal stages. A possible explanation for the variability of trunk and tail phenotype is the much larger distance of migration of the neural crest derived cells (e.g. skin melanocytes) to their target area, which could increase developmental “noise”. There is also limited variability detected in the mutant *mtl* otocyst at early postnatal stages. The appearance of the bone capsule at the vestibular pole, like a very characteristic bulb-like shape, and the length of the truncated cochlear duct and the transitional area, are slightly more variable features but always without ambiguity in comparison to littermate controls.

All *mtl* and *bsd* mutants showed a complete absence of the endolymphatic duct/sac and semicircular canals, a truncated and malformed cochlear duct and identifiable although misshaped sensory patches. We did not observe notable interaural variability in individuals, just an intrinsic range of inter-individual variability among animals with identical genotype. Despite the fact that *mtl* mice have a mixed genetic background and *bsd* mice are 129S5, the phenotype observed in the homozygotes of both strains is very robust and consistent suggesting that the phenotype is not very sensitive to the genetic background. In *Lmx1a^dr-J^* mice there is no endolymphatic duct/sac and there is a severely malformed vestibular system [13], [14]. This phenotype is very similar to the one shown by *mtl* and *bsd* homozygotes but it is unknown if the rest of the alleles reported to date [5] show the same phenotype or if there are some phenotypic traits unique to specific alleles.

### LMX1A Protein and Gene Structure are Highly Conserved in Human and Mouse

LMX1A is a LIM homeobox transcription factor located in chromosome 1 in human and mice. The protein contains 382 amino acids and is highly conserved among eutherians (NCBI-Protein). Apart from exons containing 5′ and 3′ UTRs, the exon-intron structure of the entire *Lmx1a* genomic locus is also highly conserved in human and mouse. For coding-only exons the length of the successive exons is identical in both species. The degree of conservation of splice donor and acceptor sequences is lower, but the human splice donor site of exon 4 also contains a stop codon (TAG) in the corresponding position to mouse exon 4. Protein alignment between murine and human *Lmx1a* shows 98% identity (NCBI Blast). There are no germ-line transmitted mutations in human *Lmx1a* reported to date, nor any proven link to any human disease or known syndrome. *LMX1A* function in humans might be backed up by redundancy, albeit there is no evidence of closely related functional paralogues of *Lmx1a* that would be able to compensate for *Lmx1a* loss of function. For instance, *Lmx1b* function is accounted for and described in human. Mutations in *LMX1B* are dominant due to happloinsufficiency (Nail-Patella syndrome, OMIM 161200; [44], [45]) and the mouse model also shows multiple roles for LMX1B in the development of limb, skeleton, eye, kidney and brain [46]. Given the higher number of isoforms for *LMX1A* in humans and the higher degree of complexity of the human brain, especially the cortex, it is possible that loss of LMX1A function in human development might be deleterious early in gestation. The fact that three different somatic mutations in *LMX1A* have been found in gliomas of the CNS (COSMIC,http://www.sanger.ac.uk/perl/genetics/CGP/cosmic? action = gene&ln = LMX1A) may shed light on potential (postnatal) functions of *LMX1A* in the human CNS on top of the well-researched role of this gene in midbrain and the development of dopaminergic neurons [47].

**Figure 10:**
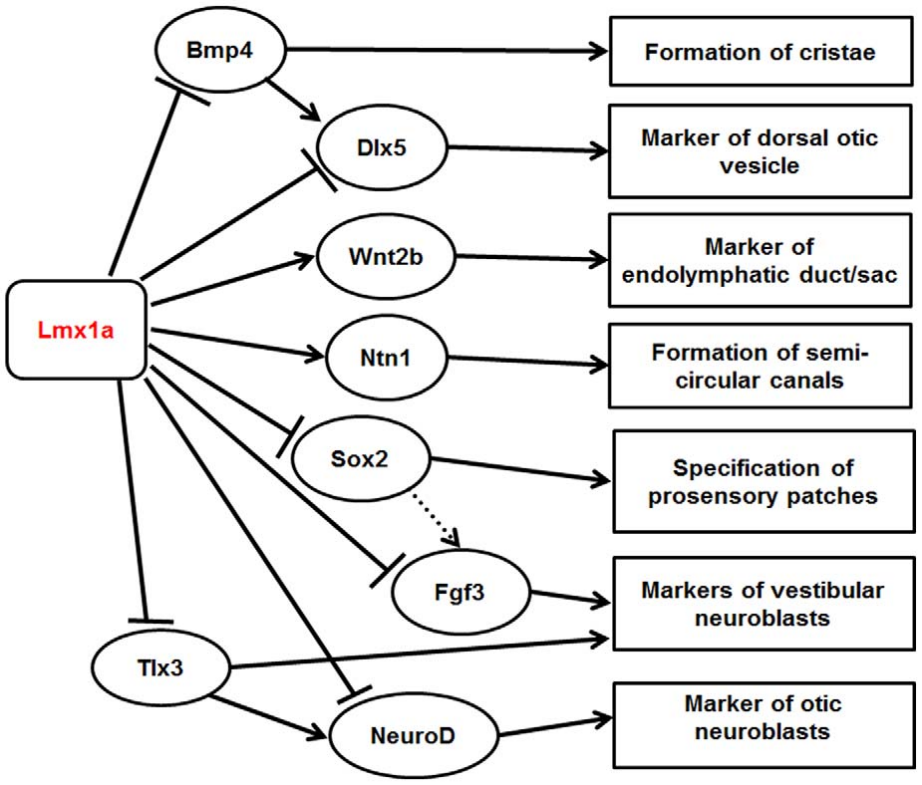
Schematic diagram showing potential interactions between Lmx1a and other markers expressed in the otocyst at around E10.5. The diagram illustrates the effects of Lmx1a on expression of eight downstream proteins: Ntn1 (this work), Sox2 [13], and Bmp4, Dlx5, Wnt2b, Fgf3, Tlx3 and NeuroD [14]. The effects were revealed by expansion or contraction of the expression domain in an *Lmx1a* mutant otocyst. Observations from early stages only were included because interpretation of expression in later stages would be confounded by the abnormality morphology of inner ear components. Interactions amongst the downstream proteins were based upon previously published data, not necessarily from the ear, identified using Ingenuity (www.ingenuity.com) [52], [53], [54], [55], [56]. All interactions may be indirect. Dotted line represents weak upregulation.

### *Lmx1a* Plays a Critical Role in Early Morphogenesis of the Inner Ear

The paint-filled ears in *mtl* and *bsd* revealed a severe malformation of the vestibule, showing a cyst without semicircular canals and no endolymphatic duct/sac. The cochlear duct was also severely malformed and truncated. Despite this abnormal morphology of the mutant inner ears the major sensory patches do develop. The hair cells in the most apical part of the truncated cochlea appear relatively normal in pattern and morphology with recognisable inner and outer hair cells and the presence of an associated tectorial membrane, although the hair cells are more disorganised than in a wildtype cochlea. This disorganisation, including extra inner hair cells, has been noted in other *Lmx1a* alleles previously [13], [14]. It is unclear if this is due to insufficient suppression of sensory fate at the edge of the prosensory patch by lack of *Lmx1a* or if this is a secondary effect due to failure of, for instance, convergent extension of the cochlear duct. In addition to this recognisable organ of Corti, we found another population of cells descending from the vestibular system towards the base of the cochlea (as reported previously [14]), which were not covered by a tectorial membrane. Taken together, our results suggest that there is no clear separation between the different sensory domains within the vestibular system and the cochlea. Our results support previous studies showing that *Lmx1a* mutants display a lack of clear segregation of sensory, non-sensory and neurogenic domains in the inner ear [13], [14]. Also, there is an abnormal enlargement of the endolymphatic space, possibly due to the lack of the endolymphatic duct/sac, which is considered critical for endolymphatic fluid homeostasis [48], [49]. Another plausible explanation for the enlargement is the failure of the semicircular canals to form and pinch off from the central region of the vestibule. These two possibilities are obviously not mutually exclusive.

We have shown that *Lmx1a* mRNA levels are significantly down-regulated in *mtl* and *bsd* mutant whole embryos at E10.5 and so are other genes, like *Hmx3*, *Hmx2*, *Netrin1* and *Pax2*. *Hmx3* and *Hmx2* knockouts show the utricle and saccule fused into a single cavity with severe malformations of the semicircular canals [35], [36], [37]. In *mtl* mutants we found complete absence of semicircular canals and we showed a significant down-regulation of *Hmx3* and *Hmx2* mRNA levels, suggesting that normal *Lmx1a* levels are required for normal expression of these transcription factors. However, the analysis of Hmx3 protein expression in sections at E10.5 showed a similar diffuse pattern in the otocyst of wildtype and *mtl* mutants, suggesting that Lmx1a is not upstream of Hmx3, and the lack of Hmx3 expression in the inner ear at E12.5 might be secondary to the lack of ED/ES and semicircular canals. Therefore the initial significant down-regulation of Hmx3 mRNA detected in whole embryos at E10.5 might be due to a down-regulation in tissues other than the ear.

*Netrin1* is expressed in the prospective fusion plate that will generate the semicircular canals [39], [50] and is also significantly down-regulated in these *Lmx1a* mutants, which lack semicircular canals, suggesting a possible requirement of *Lmx1a* for normal expression of *Netrin1* although it is not clear how directly *Lmx1a* might regulate *Netrin1*. Analysis of Netrin1 protein in wildtypes at E10.5 showed expression in the dorsolateral wall of the otocyst and in the neural tube. A similar expression pattern persists at E12.5, showing a strong expression of Netrin1 in the outpocketings of the developing semicircular canals. In *mtl* mutants Netrin1 expression is not detected in the inner ear but strong expression remains in the neural tube. These results suggest that Lmx1a is required for normal expression of Netrin1 in the inner ear.

Finally, we analysed *Pax2*, which is expressed in medial region of the mammalian otocyst partly overlapping *Lmx1a* expression at E10.5. Mutations in *Pax2* cause agenesis or severe malformation of the mouse cochlea with normal development of the vestibular region of the inner ear [41]. In *mtl* and *bsd* mutants the cochlear duct is severely truncated and under-developed, very similar to that of *Pax2* ^−/−^ mice [42], and we found a significant down-regulation of *Pax2* mRNA in these *Lmx1a* mutants at E10.5. In wildtype sections, expression of Pax2 protein was found in the otocyst at E10.5 and 2 days later strong expression was observed in the outpocketings of the semicircular canals, ED/ES and also in a more medial region of the inner ear which will develop into the cochlear domain. A similar expression pattern of Pax2 protein is observed in the otocyst of wildtypes and *mtl* mutants at E10.5, which suggest that *Lmx1a* is not required for the initial expression of Pax2. Consequently, the abnormal expression pattern of Pax2 in *mtl* mutants at E12.5 might be secondary to the lack of ED/ES and semicircular canals.

It is interesting that defects in *Hmx3*, *Hmx2* and *Netrin1* cause abnormal morphogenesis of semicircular canals and vestibular system only, whereas defects in *Pax2* lead to abnormal cochlear development without affecting vestibular system, which suggests that cochlear and vestibular systems follow independent developmental programmes. Interestingly, despite the severe malformation of the inner ear in *mtl* mutants we found that Pax2 protein expression was still detected in the presumptive developing cochlear region, whereas no Pax2 expression is found anywhere else in the inner ear due to lack of developing semicircular canals and ED/ES. This may account for the presence of a cochlea in *mtl* mutants, albeit a shortened one. These results suggest that Pax2 follows two independent developmental pathways, (1) for the specification of ED/ES and semicircular canals, and (2) for cochlear development.

*Lmx1a* mutants show defects in both cochlear and vestibular compartments suggesting that *Lmx1a* might act as an upstream regulator of key factors playing a critical role in early morphogenesis of the inner ear. We have integrated the results presented in this work and those described by Nichols *et al*. [13] and Koo *et al*. [14], to propose a network of potential interactions between *Lmx1a* and its downstream targets known to be involved in early morphogenesis of the inner ear, focussing on the early stages before abnormal morphogenesis complicates interpretation of labelling patterns (Fig. 10). In summary, these results suggest that Lmx1a represses expression of Sox2 [13], Dlx5, Bmp4, NeuroD, Tlx3 and Fgf3 [14] and activates Netrin1 (this work) and Wnt2b [14] either directly or indirectly during the development of the otic vesicle at around E10.5. Other interactions amongst the downstream proteins were based upon previously published data, not necessarily from the ear, identified using Ingenuity (www.ingenuity.com) [52], [53], [54], [55], [56].

A conditional approach, for example using the *Lmx1a^tm1a^* ^(EUCOMM)Wtsi^ or *Lmx1a^tmiTpe^* alleles, may help to address questions regarding the role of *Lmx1a* in inner ear morphogenesis, for instance, 1) if the mutant phenotype is due to defects in cell fate very early in development, perhaps even at the placode stage; 2) if the loss of endolymphatic duct formation in mutants is a consequence of a failed induction by the neural tube (which is also affected), as first suggested by Deol [51] or an autonomous inner ear defect as suggested by Koo et al [14]; 3) if defective or missing inner ear melanoblasts or other migrating neural crest cells requiring *Lmx1a* play a major role in the phenotype of *Lmx1a* mutants; and 4) if abnormal hair cell populations shown in *Lmx1a* mutants are ectopic, or switched identity or potentially both.

## Supporting Information

Figure S1 **Mapping** ***bsd*** **deletion points. A**, Diagram representing the partial genomic structure of *Lmx1* comprising exons 2 to 4 plus flanking intronic regions. Exons are represented by boxes whereas intronic regions are represented by straight lines in turn divided into 4 sectors (A–D, for intron 2–3 and E–H for intron 3–4). Pairs of primers were designed to amplify the sequences of maximum 1 Kb within these sectors (all primer sequences used for mapping these intronic regions are included in Table S3). Primers D1-15 and E1-11 were designed to map the sequence closer to exon 3 (deleted in *bsd* mutants). **B**, Table showing the results of the PCR amplification using pairs of primers described above. All genotypes (+/+, +/*bsd*, *bsd/bsd*) were tested. Those primers giving a band of the expected size were noted as yes (Y), whereas other primers failed to amplify any band at all (noted as red N). In *bsd/bsd* we failed to amplify bands the region from D4 to E7, which was considered the interval of *bsd* deletion. **C**, Table including the sequence of the primers comprising the region deleted in *bsd* mutants. The position of the primers within *Lmx1a* sequence is indicated and so is the distance of the primers from exon 3 open reading frame (ORF). The interval for *bsd* deletion was approximately 13.48 Kb.
(TIF)

Figure S2 **Origin of** ***bsd*** **mutation. A**, PCR amplification of genomic DNA from the two independent ES cell clones (clone 1 and clone 2) used for blastocyst injection and CCI18 ES cell DNA with primers specific to *Lmx1a* exon 3 plus flanking intron 4. One single band is amplified for each sample, with the expected amplicon size (450 bp). **B**, partial traces and sequence of clone 1, clone 2 and CCI18 ES cells. We found that the single nucleotide polymorphism (SNP) identified in *bsd* mutants (A to G) at 30 base pair downstream *Lmx1a* exon 4 and within intron 4–5, appears in heterozygosis (red arrowheads).
(TIF)

Table S1 **Genomic DNA.**
(PPT)

Table S2 **Transcript.**
(PPT)

Table S3 **Mapping bsd deletion.**
(PPT)
