# Supplementary figures and images for: Mutanlallemand (mtl) and Belly Spot and Deafness (bsd) Are Two New Mutations of Lmx1a Causing Severe Cochlear and Vestibular Defects
Source: PLoS One. 2012 Nov 30;7(11):e51065. doi: 10.1371/journal.pone.0051065 (PMC3511360; doi:10.1371/journal.pone.0051065)

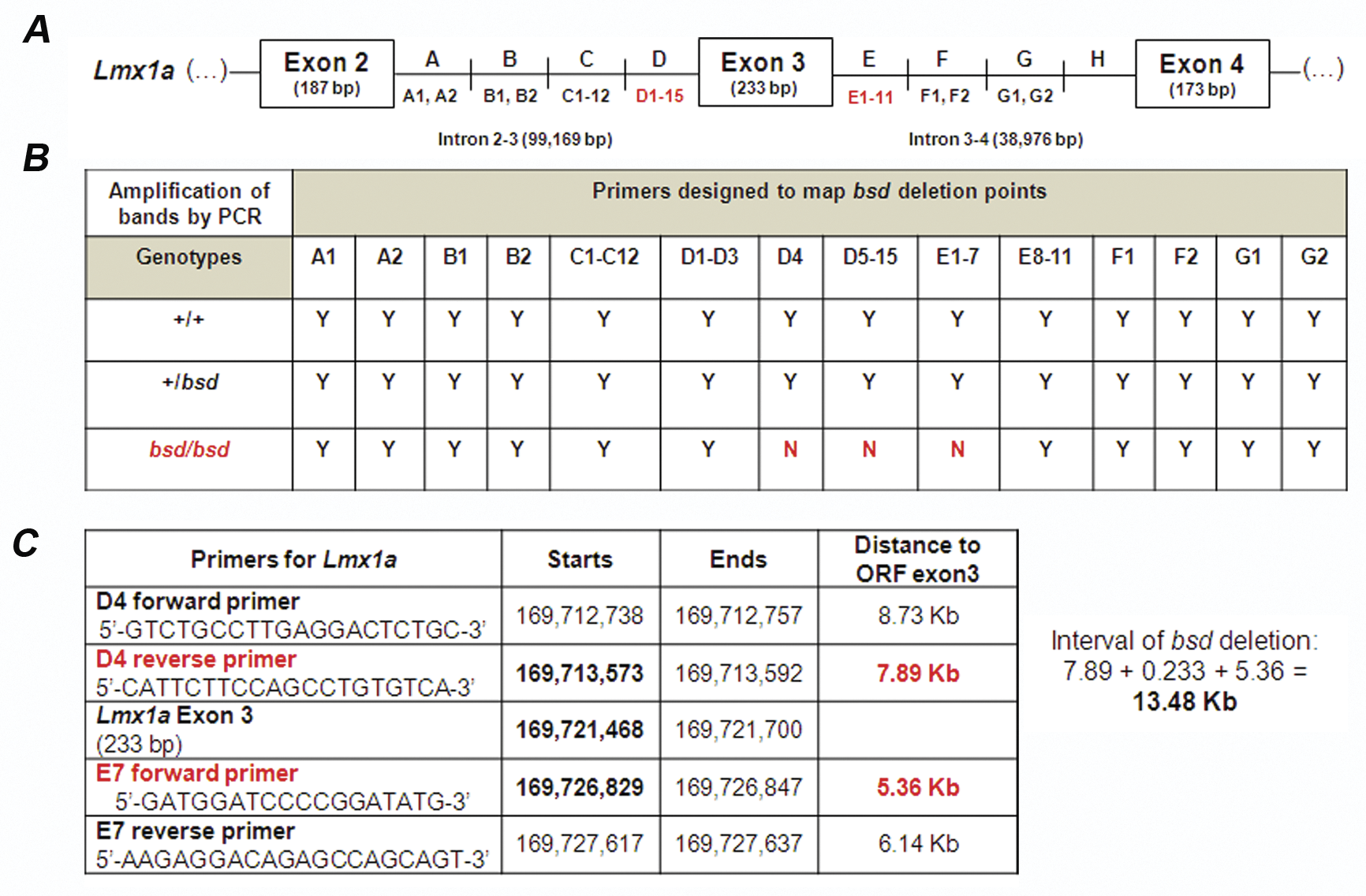

Supplement: Figure S1 — Mapping bsd deletion points. A, Diagram representing the partial genomic structure of Lmx1 comprising exons 2 to 4 plus flanking intronic regions. Exons are represented by boxes whereas intronic regions are represented by straight lines in turn divided into 4 sectors (A–D, for intron 2–3 and E–H for intron 3–4). Pairs of primers were designed to amplify the sequences of maximum 1 Kb within these sectors (all primer sequences used for mapping these intronic regions are included in Table S3). Primers D1-15 and E1-11 were designed to map the sequence closer to exon 3 (deleted in bsd mutants). B, Table showing the results of the PCR amplification using pairs of primers described above. All genotypes (+/+, +/bsd, bsd/bsd) were tested. Those primers giving a band of the expected size were noted as yes (Y), whereas other primers failed to amplify any band at all (noted as red N). In bsd/bsd we failed to amplify bands the region from D4 to E7, which was considered the interval of bsd deletion. C, Table including the sequence of the primers comprising the region deleted in bsd mutants. The position of the primers within Lmx1a sequence is indicated and so is the distance of the primers from exon 3 open reading frame (ORF). The interval for bsd deletion was approximately 13.48 Kb. (TIF) [file pone.0051065.s001.tif]

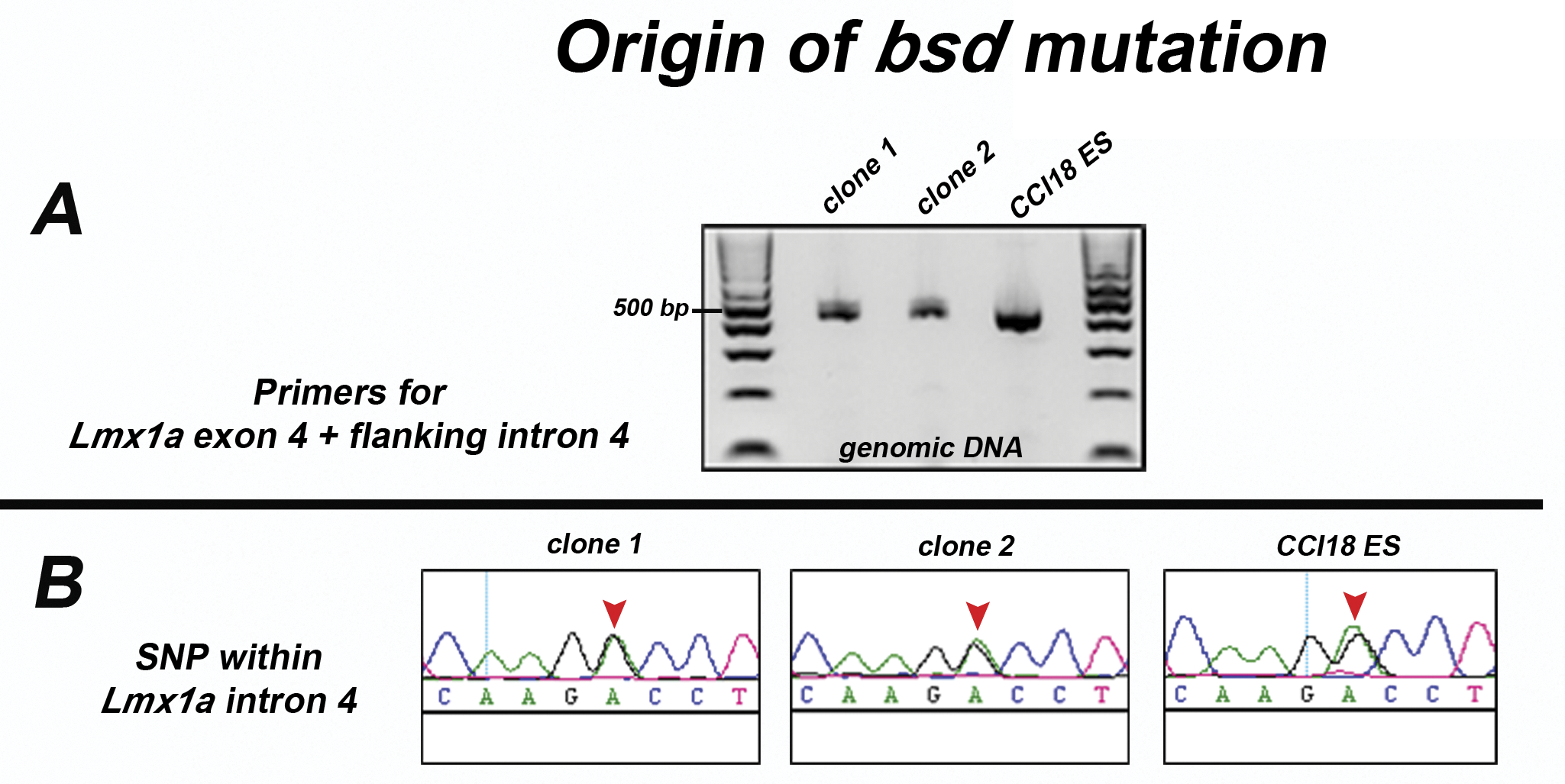

Supplement: Figure S2 — Origin of bsd mutation. A, PCR amplification of genomic DNA from the two independent ES cell clones (clone 1 and clone 2) used for blastocyst injection and CCI18 ES cell DNA with primers specific to Lmx1a exon 3 plus flanking intron 4. One single band is amplified for each sample, with the expected amplicon size (450 bp). B, partial traces and sequence of clone 1, clone 2 and CCI18 ES cells. We found that the single nucleotide polymorphism (SNP) identified in bsd mutants (A to G) at 30 base pair downstream Lmx1a exon 4 and within intron 4–5, appears in heterozygosis (red arrowheads). (TIF) [file pone.0051065.s002.tif]
