# Supplementary material for: Mutanlallemand (mtl) and Belly Spot and Deafness (bsd) Are Two New Mutations of Lmx1a Causing Severe Cochlear and Vestibular Defects
Source: PLoS One. 2012 Nov 30;7(11):e51065. doi: 10.1371/journal.pone.0051065 (PMC3511360; doi:10.1371/journal.pone.0051065)
Supplement: Table S1 — Genomic DNA. (PPT) [file pone.0051065.s003.ppt]

## Slide 1
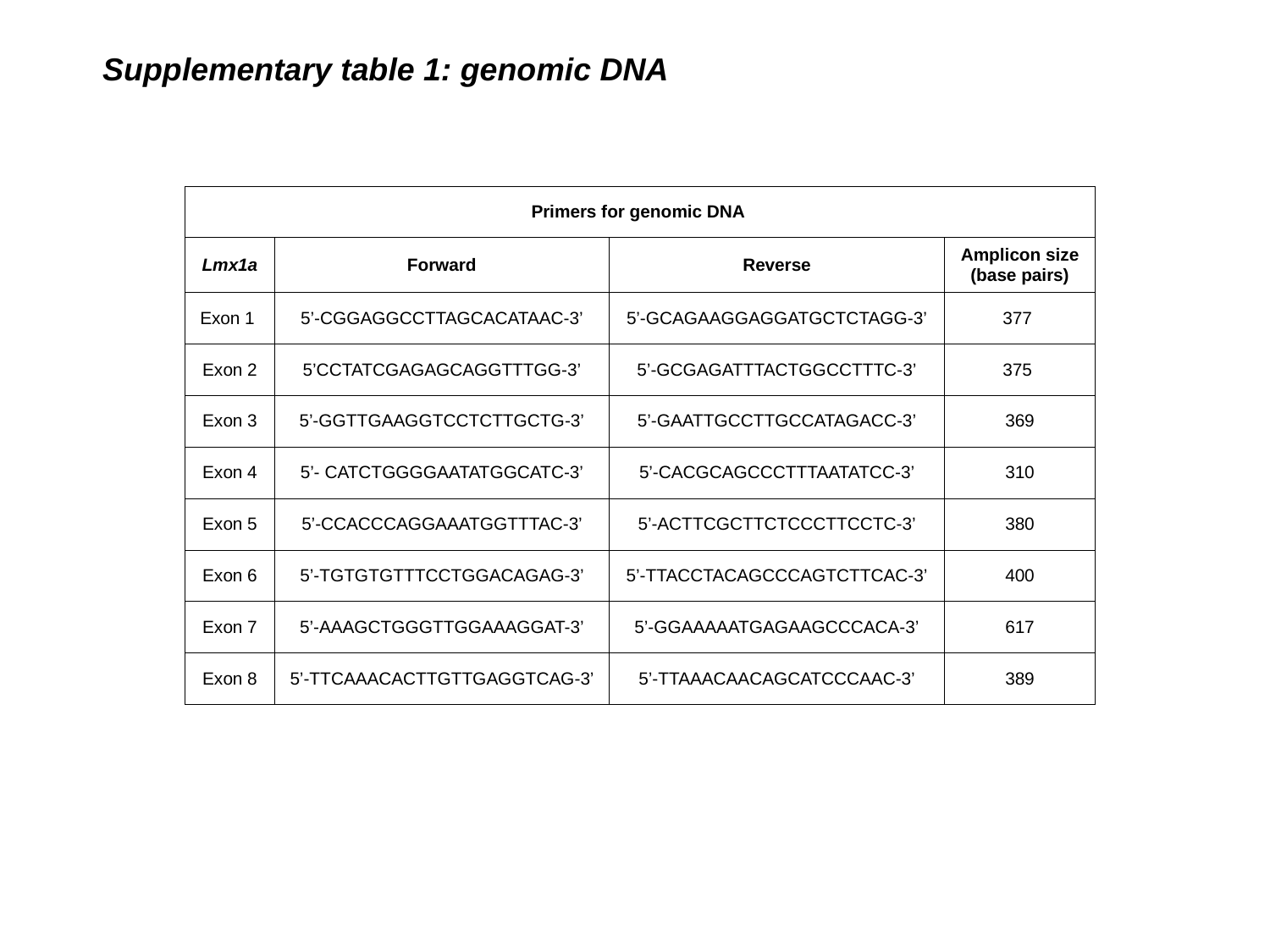

Supplementary table 1: genomic DNA
| Primers for genomic DNA | | | |
| --- | --- | --- | --- |
| Lmx1a | Forward | Reverse | Amplicon size (base pairs) |
| Exon 1 | 5’-CGGAGGCCTTAGCACATAAC-3’ | 5’-GCAGAAGGAGGATGCTCTAGG-3’ | 377 |
| Exon 2 | 5’CCTATCGAGAGCAGGTTTGG-3’ | 5’-GCGAGATTTACTGGCCTTTC-3’ | 375 |
| Exon 3 | 5’-GGTTGAAGGTCCTCTTGCTG-3’ | 5’-GAATTGCCTTGCCATAGACC-3’ | 369 |
| Exon 4 | 5’- CATCTGGGGAATATGGCATC-3’ | 5’-CACGCAGCCCTTTAATATCC-3’ | 310 |
| Exon 5 | 5’-CCACCCAGGAAATGGTTTAC-3’ | 5’-ACTTCGCTTCTCCCTTCCTC-3’ | 380 |
| Exon 6 | 5’-TGTGTGTTTCCTGGACAGAG-3’ | 5’-TTACCTACAGCCCAGTCTTCAC-3’ | 400 |
| Exon 7 | 5’-AAAGCTGGGTTGGAAAGGAT-3’ | 5’-GGAAAAATGAGAAGCCCACA-3’ | 617 |
| Exon 8 | 5’-TTCAAACACTTGTTGAGGTCAG-3’ | 5’-TTAAACAACAGCATCCCAAC-3’ | 389 |
