# Supplementary material for: Mutanlallemand (mtl) and Belly Spot and Deafness (bsd) Are Two New Mutations of Lmx1a Causing Severe Cochlear and Vestibular Defects
Source: PLoS One. 2012 Nov 30;7(11):e51065. doi: 10.1371/journal.pone.0051065 (PMC3511360; doi:10.1371/journal.pone.0051065)
Supplement: Table S2 — Transcript. (PPT) [file pone.0051065.s004.ppt]

## Slide 1
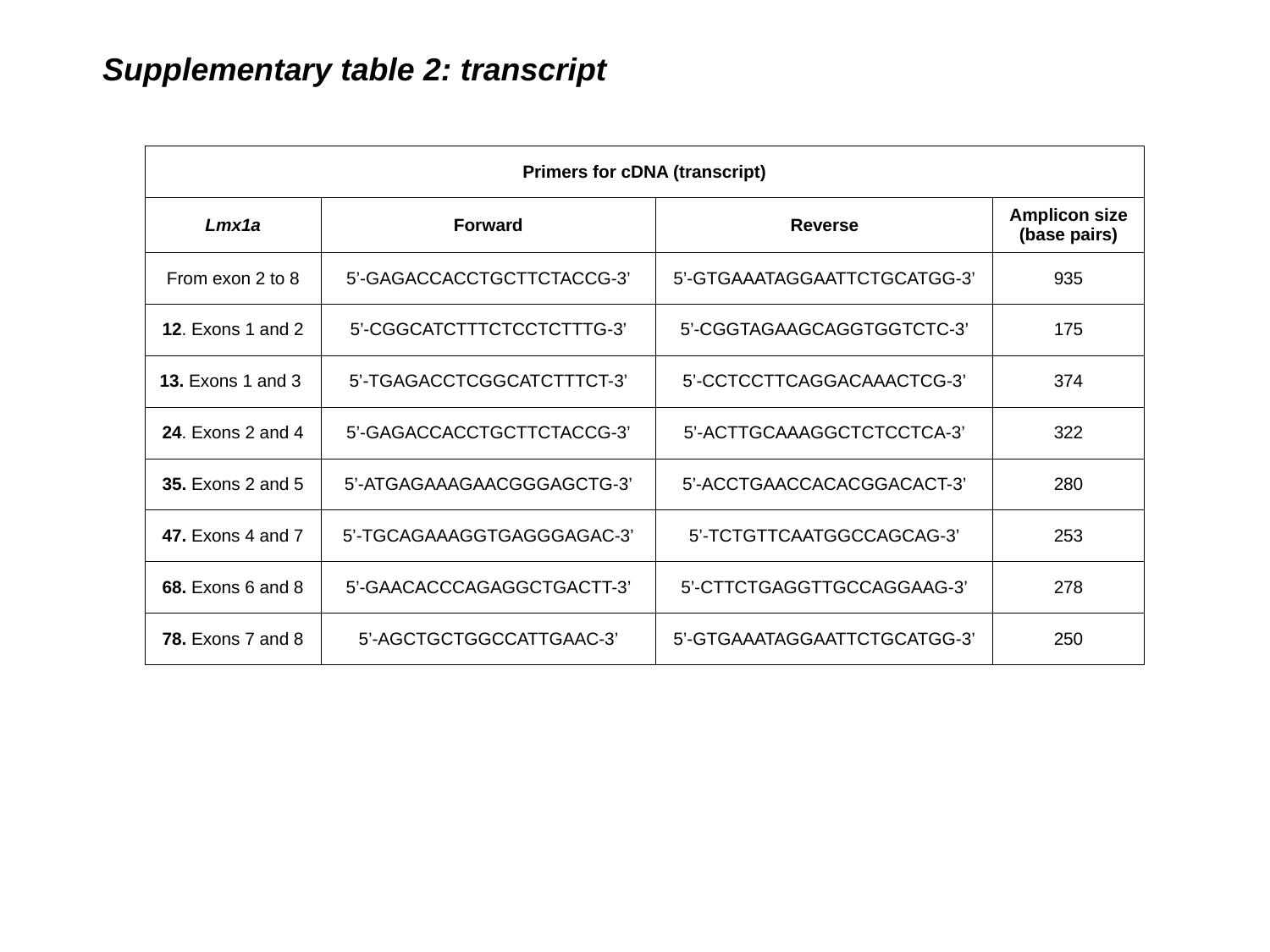

Supplementary table 2: transcript
| Primers for cDNA (transcript) | | | |
| --- | --- | --- | --- |
| Lmx1a | Forward | Reverse | Amplicon size (base pairs) |
| From exon 2 to 8 | 5’-GAGACCACCTGCTTCTACCG-3’ | 5’-GTGAAATAGGAATTCTGCATGG-3’ | 935 |
| 12. Exons 1 and 2 | 5’-CGGCATCTTTCTCCTCTTTG-3’ | 5’-CGGTAGAAGCAGGTGGTCTC-3’ | 175 |
| 13. Exons 1 and 3 | 5’-TGAGACCTCGGCATCTTTCT-3’ | 5’-CCTCCTTCAGGACAAACTCG-3’ | 374 |
| 24. Exons 2 and 4 | 5’-GAGACCACCTGCTTCTACCG-3’ | 5’-ACTTGCAAAGGCTCTCCTCA-3’ | 322 |
| 35. Exons 2 and 5 | 5’-ATGAGAAAGAACGGGAGCTG-3’ | 5’-ACCTGAACCACACGGACACT-3’ | 280 |
| 47. Exons 4 and 7 | 5’-TGCAGAAAGGTGAGGGAGAC-3’ | 5’-TCTGTTCAATGGCCAGCAG-3’ | 253 |
| 68. Exons 6 and 8 | 5’-GAACACCCAGAGGCTGACTT-3’ | 5’-CTTCTGAGGTTGCCAGGAAG-3’ | 278 |
| 78. Exons 7 and 8 | 5’-AGCTGCTGGCCATTGAAC-3’ | 5’-GTGAAATAGGAATTCTGCATGG-3’ | 250 |
