# Supplementary material for: Mutanlallemand (mtl) and Belly Spot and Deafness (bsd) Are Two New Mutations of Lmx1a Causing Severe Cochlear and Vestibular Defects
Source: PLoS One. 2012 Nov 30;7(11):e51065. doi: 10.1371/journal.pone.0051065 (PMC3511360; doi:10.1371/journal.pone.0051065)
Supplement: Table S3 — Mapping bsd deletion. (PPT) [file pone.0051065.s005.ppt]

## Slide 1
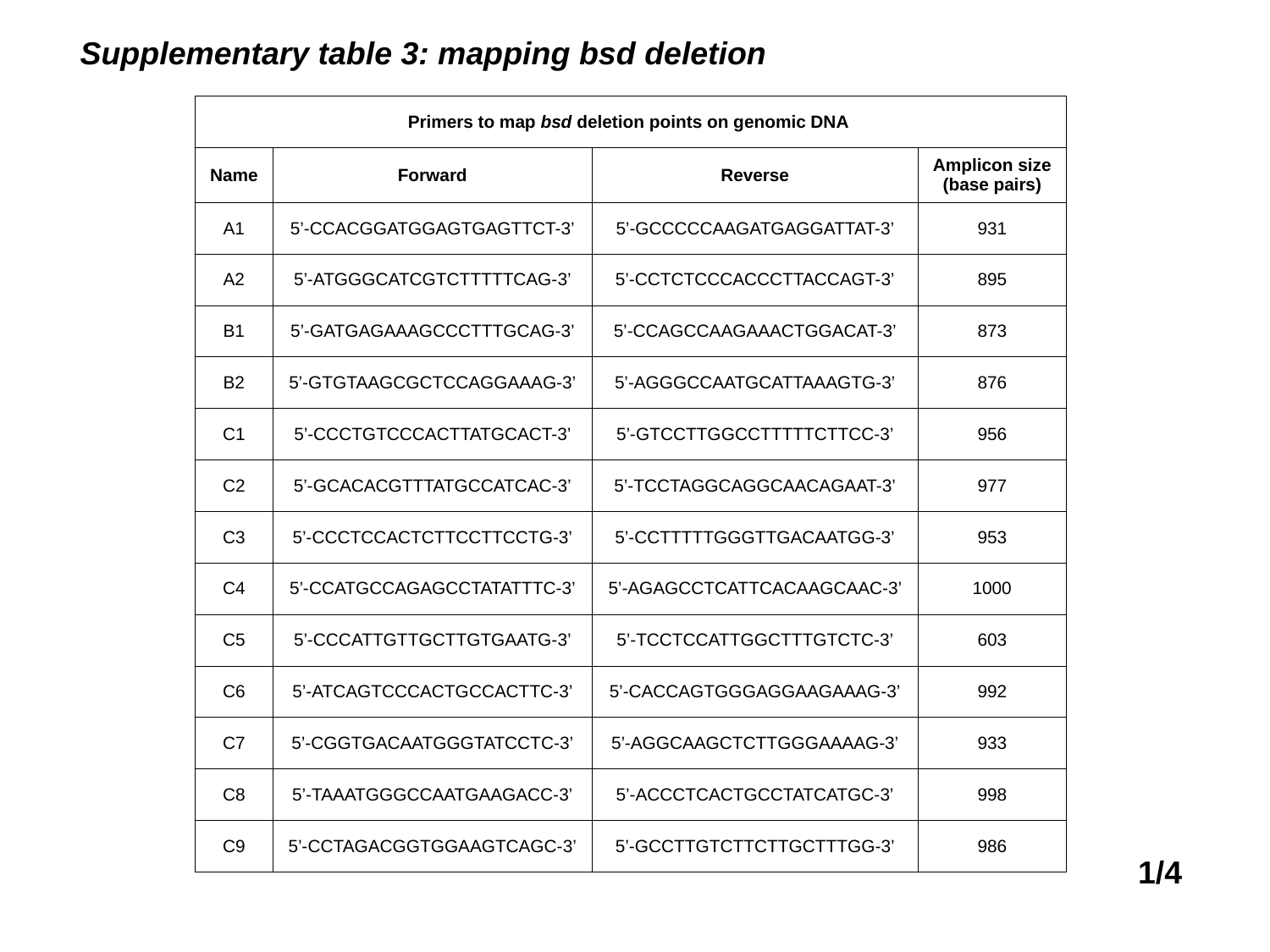

Supplementary table 3: mapping bsd deletion
| Primers to map bsd deletion points on genomic DNA | | | |
| --- | --- | --- | --- |
| Name | Forward | Reverse | Amplicon size (base pairs) |
| A1 | 5’-CCACGGATGGAGTGAGTTCT-3’ | 5’-GCCCCCAAGATGAGGATTAT-3’ | 931 |
| A2 | 5’-ATGGGCATCGTCTTTTTCAG-3’ | 5’-CCTCTCCCACCCTTACCAGT-3’ | 895 |
| B1 | 5’-GATGAGAAAGCCCTTTGCAG-3’ | 5’-CCAGCCAAGAAACTGGACAT-3’ | 873 |
| B2 | 5’-GTGTAAGCGCTCCAGGAAAG-3’ | 5’-AGGGCCAATGCATTAAAGTG-3’ | 876 |
| C1 | 5’-CCCTGTCCCACTTATGCACT-3’ | 5’-GTCCTTGGCCTTTTTCTTCC-3’ | 956 |
| C2 | 5’-GCACACGTTTATGCCATCAC-3’ | 5’-TCCTAGGCAGGCAACAGAAT-3’ | 977 |
| C3 | 5’-CCCTCCACTCTTCCTTCCTG-3’ | 5’-CCTTTTTGGGTTGACAATGG-3’ | 953 |
| C4 | 5’-CCATGCCAGAGCCTATATTTC-3’ | 5’-AGAGCCTCATTCACAAGCAAC-3’ | 1000 |
| C5 | 5’-CCCATTGTTGCTTGTGAATG-3’ | 5’-TCCTCCATTGGCTTTGTCTC-3’ | 603 |
| C6 | 5’-ATCAGTCCCACTGCCACTTC-3’ | 5’-CACCAGTGGGAGGAAGAAAG-3’ | 992 |
| C7 | 5’-CGGTGACAATGGGTATCCTC-3’ | 5’-AGGCAAGCTCTTGGGAAAAG-3’ | 933 |
| C8 | 5’-TAAATGGGCCAATGAAGACC-3’ | 5’-ACCCTCACTGCCTATCATGC-3’ | 998 |
| C9 | 5’-CCTAGACGGTGGAAGTCAGC-3’ | 5’-GCCTTGTCTTCTTGCTTTGG-3’ | 986 |
1/4

## Slide 2
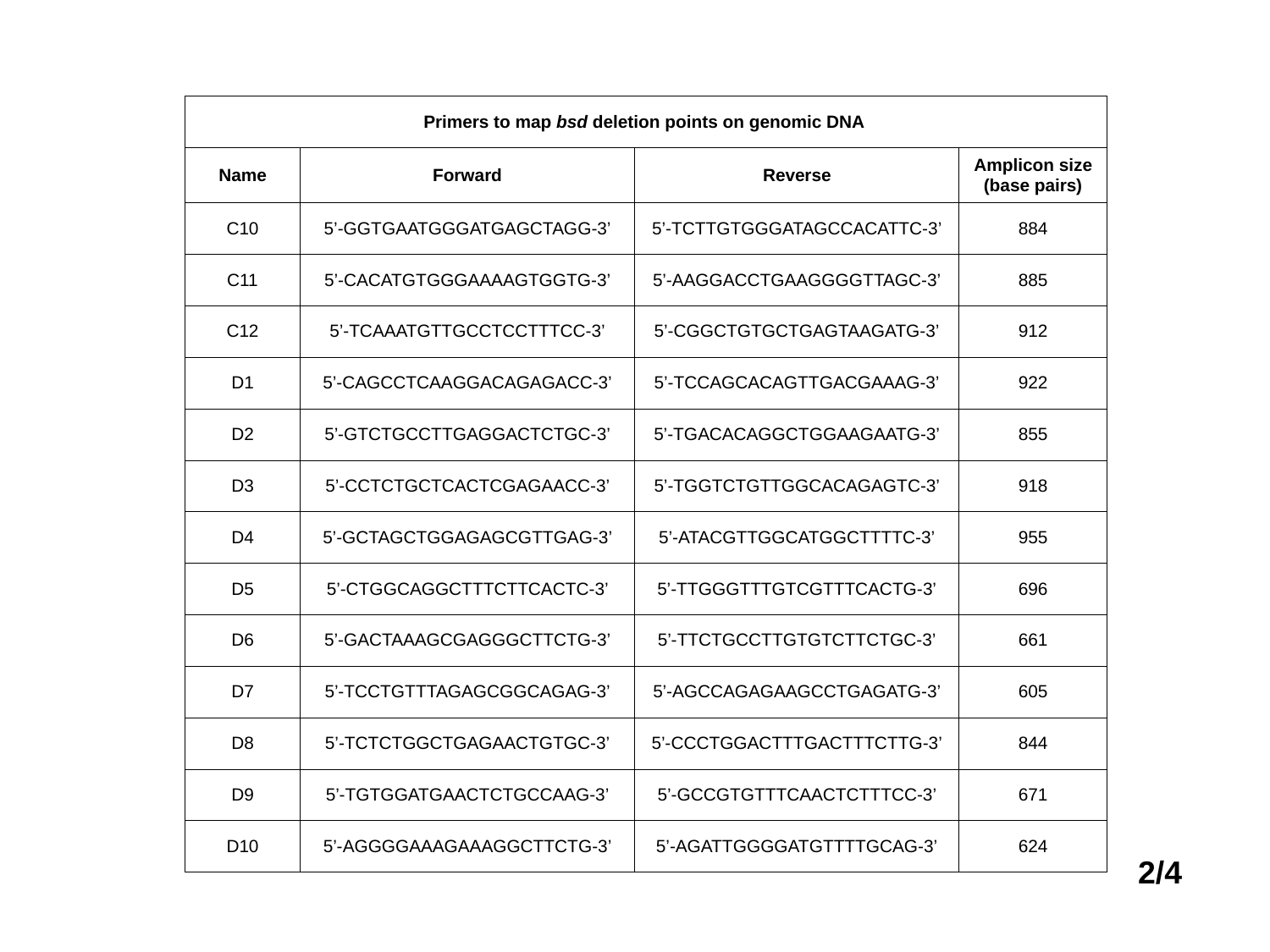

| Primers to map bsd deletion points on genomic DNA | | | |
| --- | --- | --- | --- |
| Name | Forward | Reverse | Amplicon size (base pairs) |
| C10 | 5’-GGTGAATGGGATGAGCTAGG-3’ | 5’-TCTTGTGGGATAGCCACATTC-3’ | 884 |
| C11 | 5’-CACATGTGGGAAAAGTGGTG-3’ | 5’-AAGGACCTGAAGGGGTTAGC-3’ | 885 |
| C12 | 5’-TCAAATGTTGCCTCCTTTCC-3’ | 5’-CGGCTGTGCTGAGTAAGATG-3’ | 912 |
| D1 | 5’-CAGCCTCAAGGACAGAGACC-3’ | 5’-TCCAGCACAGTTGACGAAAG-3’ | 922 |
| D2 | 5’-GTCTGCCTTGAGGACTCTGC-3’ | 5’-TGACACAGGCTGGAAGAATG-3’ | 855 |
| D3 | 5’-CCTCTGCTCACTCGAGAACC-3’ | 5’-TGGTCTGTTGGCACAGAGTC-3’ | 918 |
| D4 | 5’-GCTAGCTGGAGAGCGTTGAG-3’ | 5’-ATACGTTGGCATGGCTTTTC-3’ | 955 |
| D5 | 5’-CTGGCAGGCTTTCTTCACTC-3’ | 5’-TTGGGTTTGTCGTTTCACTG-3’ | 696 |
| D6 | 5’-GACTAAAGCGAGGGCTTCTG-3’ | 5’-TTCTGCCTTGTGTCTTCTGC-3’ | 661 |
| D7 | 5’-TCCTGTTTAGAGCGGCAGAG-3’ | 5’-AGCCAGAGAAGCCTGAGATG-3’ | 605 |
| D8 | 5’-TCTCTGGCTGAGAACTGTGC-3’ | 5’-CCCTGGACTTTGACTTTCTTG-3’ | 844 |
| D9 | 5’-TGTGGATGAACTCTGCCAAG-3’ | 5’-GCCGTGTTTCAACTCTTTCC-3’ | 671 |
| D10 | 5’-AGGGGAAAGAAAGGCTTCTG-3’ | 5’-AGATTGGGGATGTTTTGCAG-3’ | 624 |
2/4

## Slide 3
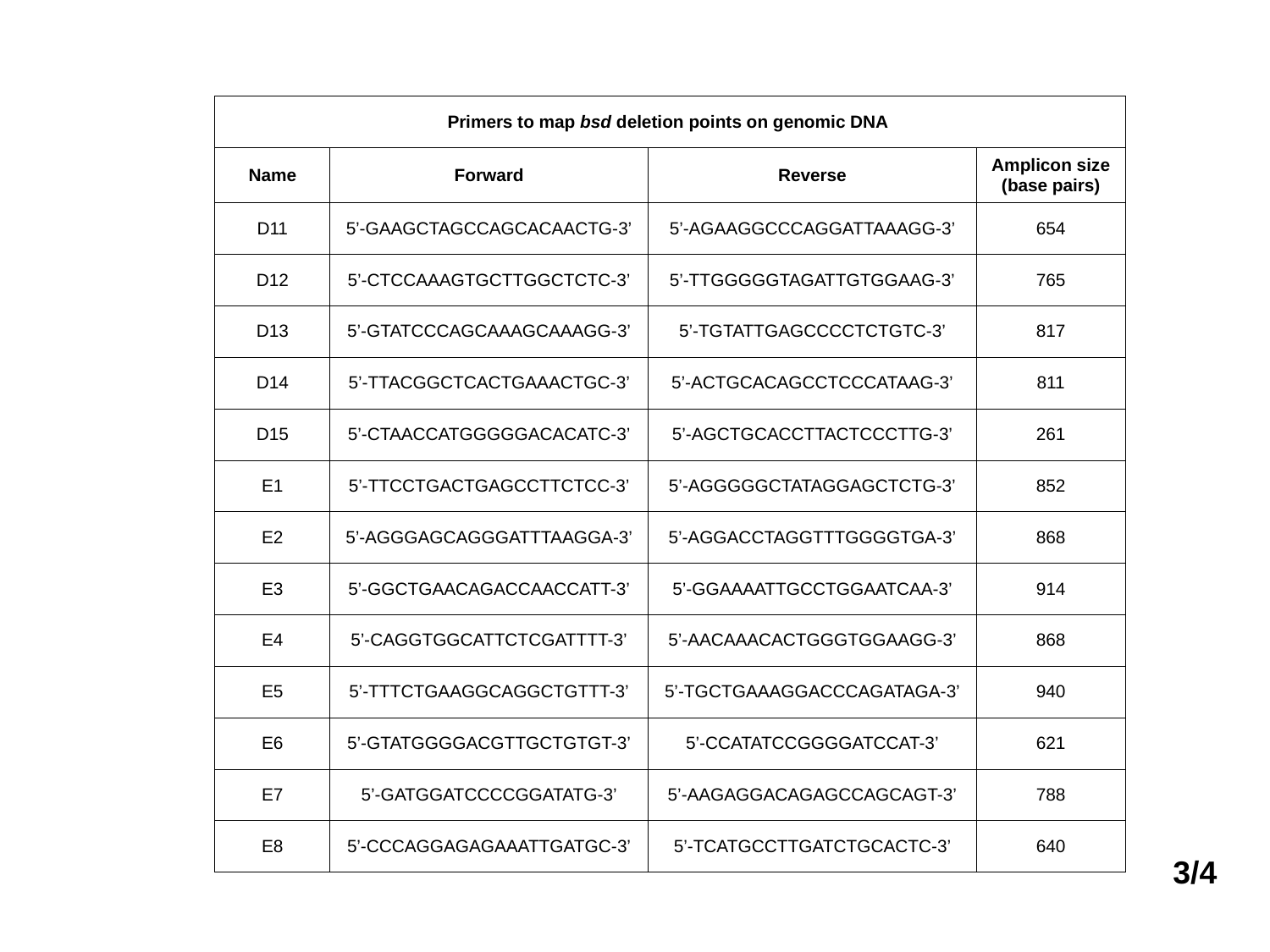

| Primers to map bsd deletion points on genomic DNA | | | |
| --- | --- | --- | --- |
| Name | Forward | Reverse | Amplicon size (base pairs) |
| D11 | 5’-GAAGCTAGCCAGCACAACTG-3’ | 5’-AGAAGGCCCAGGATTAAAGG-3’ | 654 |
| D12 | 5’-CTCCAAAGTGCTTGGCTCTC-3’ | 5’-TTGGGGGTAGATTGTGGAAG-3’ | 765 |
| D13 | 5’-GTATCCCAGCAAAGCAAAGG-3’ | 5’-TGTATTGAGCCCCTCTGTC-3’ | 817 |
| D14 | 5’-TTACGGCTCACTGAAACTGC-3’ | 5’-ACTGCACAGCCTCCCATAAG-3’ | 811 |
| D15 | 5’-CTAACCATGGGGGACACATC-3’ | 5’-AGCTGCACCTTACTCCCTTG-3’ | 261 |
| E1 | 5’-TTCCTGACTGAGCCTTCTCC-3’ | 5’-AGGGGGCTATAGGAGCTCTG-3’ | 852 |
| E2 | 5’-AGGGAGCAGGGATTTAAGGA-3’ | 5’-AGGACCTAGGTTTGGGGTGA-3’ | 868 |
| E3 | 5’-GGCTGAACAGACCAACCATT-3’ | 5’-GGAAAATTGCCTGGAATCAA-3’ | 914 |
| E4 | 5’-CAGGTGGCATTCTCGATTTT-3’ | 5’-AACAAACACTGGGTGGAAGG-3’ | 868 |
| E5 | 5’-TTTCTGAAGGCAGGCTGTTT-3’ | 5’-TGCTGAAAGGACCCAGATAGA-3’ | 940 |
| E6 | 5’-GTATGGGGACGTTGCTGTGT-3’ | 5’-CCATATCCGGGGATCCAT-3’ | 621 |
| E7 | 5’-GATGGATCCCCGGATATG-3’ | 5’-AAGAGGACAGAGCCAGCAGT-3’ | 788 |
| E8 | 5’-CCCAGGAGAGAAATTGATGC-3’ | 5’-TCATGCCTTGATCTGCACTC-3’ | 640 |
3/4

## Slide 4
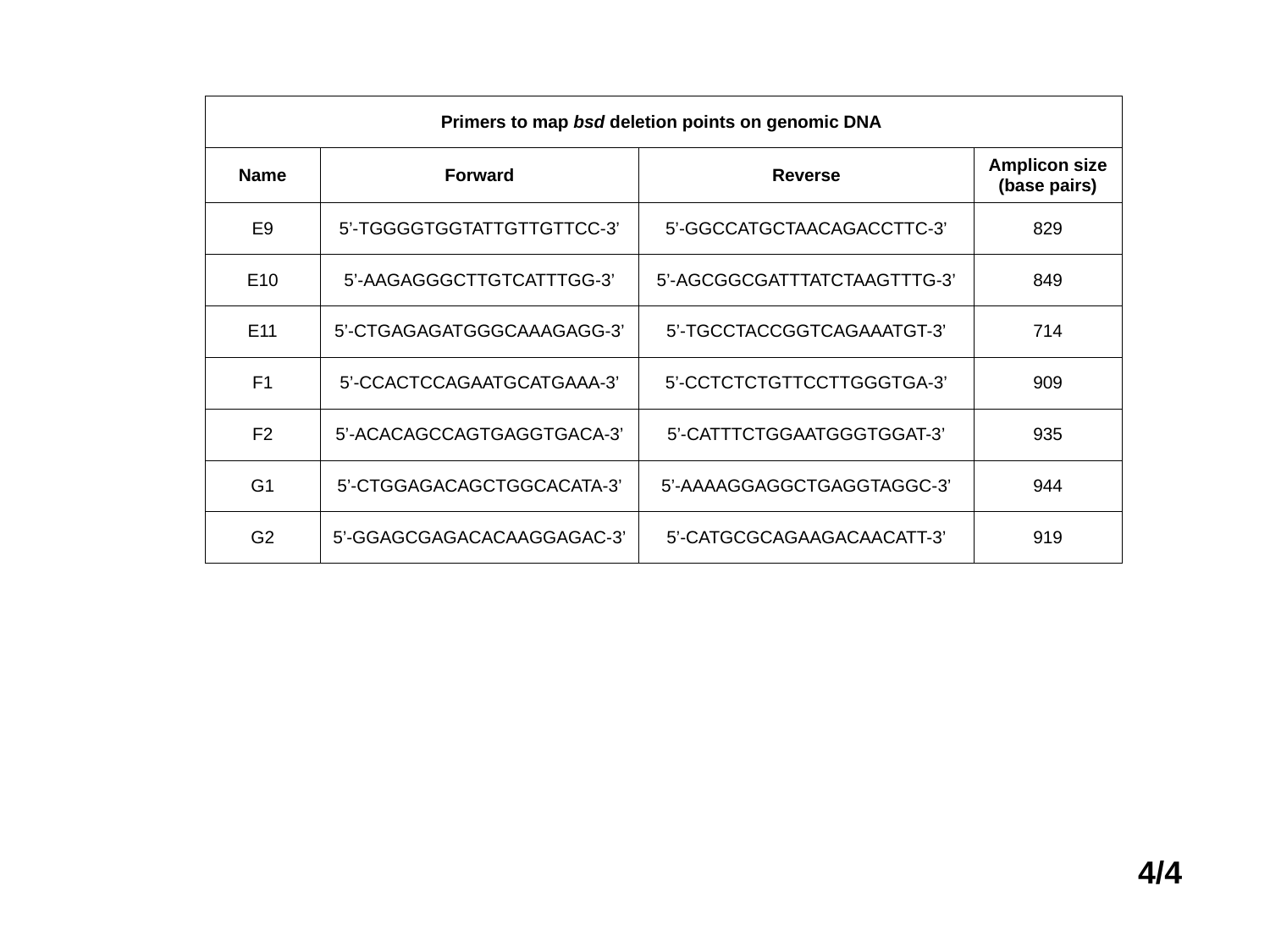

| Primers to map bsd deletion points on genomic DNA | | | |
| --- | --- | --- | --- |
| Name | Forward | Reverse | Amplicon size (base pairs) |
| E9 | 5’-TGGGGTGGTATTGTTGTTCC-3’ | 5’-GGCCATGCTAACAGACCTTC-3’ | 829 |
| E10 | 5’-AAGAGGGCTTGTCATTTGG-3’ | 5’-AGCGGCGATTTATCTAAGTTTG-3’ | 849 |
| E11 | 5’-CTGAGAGATGGGCAAAGAGG-3’ | 5’-TGCCTACCGGTCAGAAATGT-3’ | 714 |
| F1 | 5’-CCACTCCAGAATGCATGAAA-3’ | 5’-CCTCTCTGTTCCTTGGGTGA-3’ | 909 |
| F2 | 5’-ACACAGCCAGTGAGGTGACA-3’ | 5’-CATTTCTGGAATGGGTGGAT-3’ | 935 |
| G1 | 5’-CTGGAGACAGCTGGCACATA-3’ | 5’-AAAAGGAGGCTGAGGTAGGC-3’ | 944 |
| G2 | 5’-GGAGCGAGACACAAGGAGAC-3’ | 5’-CATGCGCAGAAGACAACATT-3’ | 919 |
4/4
